# Improved outcome for AML patients over the years 2000–2014

**DOI:** 10.1038/s41408-017-0011-1

**Published:** 2017-11-29

**Authors:** Sarah Bertoli, Suzanne Tavitian, Anne Huynh, Cécile Borel, Sarah Guenounou, Isabelle Luquet, Eric Delabesse, Audrey Sarry, Guy Laurent, Michel Attal, Françoise Huguet, Emilie Bérard, Christian Récher

**Affiliations:** 1 Service d’Hématologie, Centre Hospitalier Universitaire de Toulouse, Institut Universitaire du Cancer de Toulouse Oncopole, Toulouse, France; 2 Université Toulouse III Paul Sabatier, Toulouse, France; 3 Cancer Research Center of Toulouse, UMR1037-INSERM, ERL5294 CNRS, Toulouse, France; 4 Laboratoire d’Hématologie, Centre Hospitalier Universitaire de Toulouse, Institut Universitaire du Cancer de Toulouse Oncopole, Toulouse, France; 5 Service d’Epidémiologie, Centre Hospitalier Universitaire de Toulouse, Toulouse, France; 6 UMR 1027, INSERM-Université de Toulouse III, Toulouse, France

## Abstract

Few recent studies from registries have reported an improvement in overall survival of younger patients with acute myeloid leukemia (AML). However, reasons for this improvement are not defined. We analyzed the therapeutic course and outcome of 976 patients treated by intensive chemotherapy between 2000 and 2014. The number of patients receiving allogeneic stem cell transplantation in first or second response significantly increased over time whereas autologous transplantation was progressively abandoned. In the 513 younger patients, there were no differences in first complete response, induction failure, incidence of relapse, or non-relapse mortality over time. The period of time was significantly associated with a better overall survival especially in 2010–2014. The 2010–2014 period effect was still significant in multivariate analysis and was independent of allogeneic stem cell transplantation. In the 463 older patients, there was a significant interaction between the period and leukocytosis in multivariate analysis meaning that the 2010–2014 period had only an impact in patients with white blood cell count >50 giga/L for response and overall survival. Progresses have been made in each phase of the therapeutic course of younger AML patients resulting in survival improvement. In older patients, the outcome of hyperleukocytic patients has significantly improved in 2010–2014.

## Introduction

Acute myeloid leukemia (AML) is a hematological malignancy in which no key specific treatment has been approved compared to the progress made with multiple myeloma, B-cell lymphoma, chronic myelocytic leukemia, or acute lymphoblastic leukemia, which all recently benefited from the registration of very potent drugs^1, 2^. As far as younger and fit older AML patients are concerned, the therapeutic strategy has remained unchanged for decades based on the so called “7 + 3” induction chemotherapy using continuous-infusion cytarabine with an anthracycline. In patients having achieved complete remission, standard post remission treatments include chemotherapy as well as hematopoietic stem cell transplantation. Decision making for allogeneic transplantation relies on the probability of relapse, better predicted by the AML genetic risk and on the risk of treatment-related death predicted by specific scorings^3^.

Yet, few recent studies from registries or compilation of clinical trials have reported an improvement in overall survival (OS) of adult AML patients, especially in patients of 60 years of age or younger^4–8^. Population-based studies from registries can provide a more complete assessment of progress in survival than is available from clinical trials, which often preselect younger and generally healthier subjects and may not be representative of the average diagnosed individual. Studying survival trends over time can provide information about cancer treatment and control efforts. However, though these studies deal with large numbers, variable provided remains scarce (i.e., age, sex, and survival) and reasons for this improvement are not clearly defined. Such reasons for improvement are likely to be multiple as the road to cure for AML patients is a multi-step process subjected to numerous events and adverse effects, as well as various treatment options, especially with regards to allogeneic stem cell transplantation allocation or access to clinical trials. Although there has been no improvement in “7 + 3” induction using a third drug to treat AML, despite many attempts, many other key points of the treatments have evolved over time in current practice especially with respects to supportive care, stratification of treatment according to genetic risk and allogeneic stem cell transplantation indications.

We analyzed the main changes in routine practice and the outcome of AML patients treated between 2000 and 2014 by intensive chemotherapy in order to determine whether there has been an improvement in survival over time and if this improvement is independent of standard prognostic factors.

## Subjects and methods

### Patients

Between January 2000 and December 2014, a total of 976 patients with a newly diagnosed AML were consecutively treated with intensive chemotherapy at Toulouse University Hospital. The leukemia unit of the Toulouse University Hospital is the only certified center for the treatment of AML in the Midi-Pyrénées region (Southwest of France), which contains 3 million inhabitants. Patients are referred by personal physicians or primary care centers and are firstly seen by leukemia specialists either as outpatients for rapid diagnosis and work-up or directly as in patients if urgent medical interventions are needed. Patients from the Midi-Pyrénées area are recorded each week in the leukemia unit according to guidelines from the Oncomip network (http://www.oncomip.org) after informed consent. Patients were included in this study if they received at least one dose of intensive chemotherapy regimen. Patients with acute promyelocytic leukemia were not considered. The cytogenetic classification was in accordance with the Medical Research Council (MRC) classification^9^. Molecular analyses were performed at diagnosis or retrospectively from samples stored in the tumor bank of the U1037 Inserm department (no. DC-2008-307-CPTP1 HIMIP)^10^. Data were collected from the patients’ files and certified by the Data Management Committee of the anonymized AML database of Toulouse University Hospital and registered at the *Commission Nationale de l’Informatique et des Libertés* (CNIL) under access No. 1778920. In accordance with the Declaration of Helsinki, the study was reviewed and approved by the research ethics committee at Toulouse University Hospital.

### Treatments

Patients received intensive induction chemotherapy that included daunorubicin or idarubicin together with cytarabine either by continuous intravenous or bolus infusion as part of, or according to French clinical trials for younger and older (>60 years) AML patients^11–16^. Hydroxyurea could be given promptly at diagnosis for white blood cell (WBC) reduction in hyperleukocytic patients. Leukapheresis were not performed in our institution. Responding patients with intermediate or high risk AML and a matched sibling or 10/10 HLA allele fully matched unrelated donor were allocated to allogeneic stem cell transplantation. Patients with no HLA-matched donor received a consolidation regimen consisted of high-dose cytarabine followed by autologous stem-cell transplantation or three courses of high-dose cytarabine. After achieving complete response (CR), patients over 60 received maintenance therapy with idarubicin and low-dose cytarabine. Refractory or relapsed patients could receive salvage therapy using several chemotherapy regimens of different dose-intensity that are described in Supplementary Methods. Supportive care that included treatment of febrile neutropenia and disseminated intravascular coagulopathy, as well as blood-product transfusions, were given according to guidelines, which did not change over the study period and were in accordance with standard recommendations^17^. CR referred to the combination of CR and CR with incomplete blood count recovery (CRi) after one or two induction cycles were defined by the international consensus criteria^17^.

Conditioning regimens for allogeneic stem cell transplantation were classified according to a consensus definition of conditioning regimen intensity^18^. Myeloablative conditioning consisted of high-dose cyclophosphamide with total-body irradiation or busulfan. Reduced-intensity and non-myeloablative conditioning were grouped together under the reduced-intensity conditioning which associated busulfan, fludarabine and antithymocyte globulin or fludarabine and low total-body irradiation. Graft vs. host disease prevention consisted in cyclosporine alone or in combination with either methotrexate or mycophenolate mofetil. Conditioning regimens for autologous stem cell transplantation have been published elsewhere^19^.

### Outcomes

Primary outcome was OS. Secondary outcomes were day-60 death (to the date of diagnosis), first and second CR, induction failure, cumulative incidence of relapse (CIR), non-relapse mortality (NRM), and disease-free survival (DFS)^17^. CIR was measured only for patients achieving CR, from the date of remission achievement to the date of relapse. NRM was treated as competing events of relapse and patients not known to have died at last follow-up are censored on the date they were last known to be alive. DFS was measured only for patients achieving CR, from the date of remission achievement to the date of relapse or death from any cause; patients not known to have died at last follow-up are censored on the date they were last known to be alive.

### Statistical analyses

Since therapeutic strategies of AML differed between younger and older patients, we analyzed separately the outcome of patients <60 years (*n* = 513) and patients aged 60 years or older (*n* = 463) during the 2000–2004, 2005–2009, and 2010–2014 periods. We first described patients’ characteristics at diagnosis using number and frequency for qualitative data; median and interquartile range (IQR) for quantitative data. Differences in day-60 death, response, and induction failure according to the periods were tested in univariate analyses using *χ* ^2^ test (or Fisher’s exact test in case of small expected numbers). Multivariate analyses were conducted using logistic regression. For univariate survival analyses of OS and DFS, Kaplan–Meier survival curves were drawn and differences in survival functions were tested using the Log-Rank test. Univariate survival analyses used Cumulative Incidence Functions and Gray’s test for CIR and NRM, since NRM and relapse were treated as competing events. Adjusted hazard ratios (HR) and 95% confidence intervals (CI) were assessed using a standard Cox model, for OS and DFS, and a proportional sub distribution hazard model which is an extension of the Cox model for the situation of competing risks, for CIR and NRM^20^. Multivariate analyses initially included periods together with potential confounding factors (age, WBC count, AML status, cytogenetic risk, and allogeneic stem-cell transplantation for OS, DFS, and CIR). Then we used stepwise regression to assess variables that were significantly and independently associated with endpoints (*P* < 0.05). The proportional-hazard assumption was tested for each covariate of the Cox model by the “log–log” plot method curves and was always met. When linearity hypothesis was not respected, continuous potential confounding factors were transformed into ordered data. Interactions between the period and independent covariates were tested in final models. Allogeneic stem-cell transplantation was evaluated as time-dependent covariate. In order to compare patients with homogeneous maximal follow-up, patients in the first and second period were censored at 7 years. All reported *P*-values were two-sided and the significance threshold was <0.05. Statistical analyses were performed on STATA® version 14.1 (STATA Corp., College Station, TX).

## Results

### Main changes in routine practice during the study period

Chemotherapy regimens used in younger patients (<60 years) during the study period, as well as doses of anthracyclines and cytarabine, are shown in Supplementary Table 1. Anthracyclines were no longer used in consolidation from 2010. Although anthracycline dose-intensification during induction with either daunorubicin (270 mg/m^2^) or idarubicin (45 mg/m^2^) started as soon as 2010, the cumulative dose of daunorubicin or idarubicin was lower in the 2010–2014 period. In older patients (≥60 years), idarubicin and cytarabine doses did not change during the study period at both induction and consolidation^21^. With regards to prophylaxis of invasive fungal infections during induction chemotherapy, fluconazole was used in the earlier period followed by voriconazole or caspofungin in 2003 and then, posaconazole in 2008^22, 23^. Starting from 2010, dexamethasone (10 mg b.i.d, 3 days) was added to induction chemotherapy in patients with WBC > 100 giga/L or in patients with >50 giga/L and symptoms of leukostasis^24^. Indications for allogeneic stem cell transplantation in first CR evolved from geno-identical to pheno-identical (from 2007) and anecdotally umbilical cord blood (from 2005), whereas autologous stem cell transplantation was progressively abandoned. In older patients, the first allogeneic stem cell transplantation with reduced-intensity regimen was performed in 2002 in patients of 50–60 years and in 2007 in patients older than 60. Molecular stratification for allogeneic stem cell transplantation indications based on *NPM1*, *FLT3-ITD*, and *CEBPA* mutations in patients with intermediate-cytogenetic risk was introduced in 2006. From this date, patients with favorable molecular risk (i.e., mutation of *NPM1* or *CEBPA* without *FLT3-ITD* mutation) were no longer allocated to allogeneic transplantation in first CR and received three cycles of high-dose cytarabine as post remission therapy or an autologous transplantation^25^. A specific unit dedicated to acute leukemia was created in the Hematology department in 2006.

### Outcome of the whole cohort

Outcome of the whole population stratified on main prognostic factors (cytogenetics, de novo vs. secondary or therapy-related AML, mutational status) during to the 2000–2004, 2005–2009, and 2010–2014 periods are shown in Supplementary Fig. 1. However, since therapeutic strategies differed between younger and older patients, we analyzed separately the outcome of patients <60 years and patients ≥60 years during the three periods.

### Outcome of younger (<60 years) patients

**Fig. 1:**
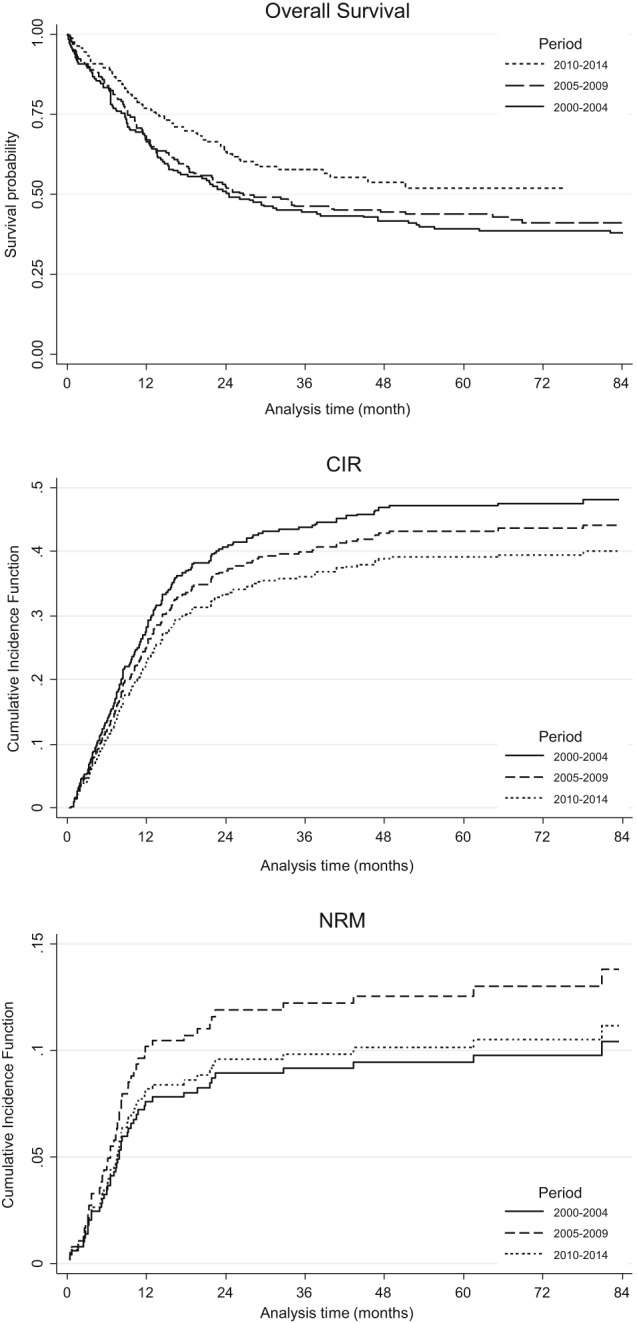
Overall survival (OS), cumulative incidence of relapse (CIR) and non-relapse mortality (NRM) in first complete response in younger (<60 years) AML patients

The characteristics of the 513 younger patients including age, AML status (de novo vs. secondary), performance status, WBC count, cytogenetic risk and *FLT3*-ITD, *NPM1*, or *CEBPA* mutations in intermediate-cytogenetic risk were well balanced during the study period (Table 1). Median follow-up of patients still alive was 67.4 months (84.0, 74.1, and 38.1 months for 2000–2004, 2005–2009, and 2010–2014, respectively). Table 2 shows response to induction, treatment distribution and main outcomes. During the induction phase, the 60-day death rate was reduced from 9.2% in 2000–2004 to 4.4% in 2010–2014 (*P* = 0.207) whereas CR and induction failure rates were similar across the study periods. In the post remission phase, the number of patients receiving allogeneic stem cell transplantation gradually increased in a significant manner (30.5 vs. 38.5% vs. 51.8% in 2000–2004, 2005–2009, and 2010–2014 periods, respectively; *P* = 0.001). This holds true for relapsed patients who achieved a second CR (*P* < 0.001). By contrast, the rate of autologous stem cell transplantation dramatically decreased (29.1 vs. 2.2% in 2000–2004 and 2010–2014 periods, respectively; *P* < 0.001). In the whole cohort of patients in first CR, the cumulative incidence of death without relapse (i.e., NRM) was 12% (95% CI, 0.09–0.15) and was not significantly associated with the period (*P* = 0.659) in univariate analysis (Fig. 1). The cumulative incidence of death without relapse in patients allografted in first CR (*n* = 171) was 20% (95% CI, 0.14–0.27) and was not significantly associated with the period (*P* = 0.940). The CIR was 44% (95% CI, 39–49) with a trend to decrease over time (HR 0.89, 95% CI, 0.63–1.26; *P* = 0.498 and HR 0.78, 95% CI, 0.54–1.11; *P* = 0.169, for 2005–2009 and 2010–2014 periods as compared to 2000–2004, respectively) (Fig. 1). Multivariate analyses with regards to 60-day induction death (HR 0.43, 95% CI, 0.17–1.13; *P* = 0.089) and DFS (HR 0.76, 95% CI 0.54–1.06; *P* = 0.104) showed a trend for a better outcome in the 2010–2014 period than in 2000–2004 (Supplementary Tables 2 and 3). Univariate analysis showed that the period of time was significantly associated with a better OS (*P* = 0.031) with HR of 0.92 (95% CI 0.70–1.20; *P* = 0.536) and 0.68 (95% CI, 0.50–0.92; *P* = 0.012) for 2005–2009 and 2010–2014, respectively, compared to 2000–2004 (Fig. 1). The 2010–2014 period effect was still significant in multivariate analysis after adjustment for age (< vs. ≥50 years), AML status (de novo vs. secondary), cytogenetics, WBC (≤ vs. > 50 giga/L) and allogeneic stem cell transplantation in CR1 (HR 0.66, 95% CI 0.49–0.91; *P* = 0.011) (Table 3). There was no significant interaction between the study period and the other independent variables in the final model indicating that the period effect was not significantly different according to WBC count, AML status, age, cytogenetic risk, or allogeneic stem cell transplantation in CR1. However, to illustrate the impact of the study period according to cytogenetics risk, WBC count, age (< vs. ≥50 years), and AML status (de novo vs. secondary), Kaplan–Meier survival curves are shown in Supplementary Fig. 2 A–D.

**Table 1:** Characteristics of younger (<60 years) AML patients

	2000–2004	2005–2009	2010–2014	Total
	*N* = 173 (33.7%)	*N* = 181 (35.3%)	*N* = 159 (31.0%)	*N* = 513 (100%)
Follow-up^a^				
Median, months (IQR)	84.0 (84.0–84.0)	74.1 (61.3–84.0)	38.1 (26.4–54.4)	67.4 (42.9–84)
Male, *n* (%)	94 (54.3)	86 (47.5)	86 (54.1)	266 (51.9)
Female, *n* (%)	79 (45.7)	95 (52.5)	73 (45.9)	247 (48.1)
Age, years (y)				
Median (IQR)	47.9 (36.4–54.5)	47.3 (37.3–55.6)	50.5 (35.7–55.7)	48.1 (36.6–55.0)
<50 years, *n* (%)	103 (59.5)	104 (57.5)	78 (49.1)	285 (55.6)
≥50 years, *n* (%)	70 (40.5)	77 (42.5)	81 (50.9)	228 (44.4)
AML status, *n* (%)				
De novo	139 (80.3)	148 (81.8)	134 (84.3)	421 (82.1)
Secondary	34 (19.7)	33(18.2)	25 (15.7)	92 (17.9)
Performance status, *n* (%)				
0–1	95 (77.2)	128 (89.5)	113 (75.8)	336 (81.0)
2–4	28 (22.8)	15 (10.5)	36 (24.2)	79 (19.0)
WBC, (giga/L)				
Median, IQR	11 (3.3–46.3)	13.3 (3.3–41)	9 (2.7–45.8)	10.8 (3.1–43.5)
≤50, *n* (%)	130 (75.6)	145 (80.1)	121 (76.1)	396 (77.3)
>50, *n* (%)	42 (24.4)	36 (19.9)	38 (23.9)	116 (22.7)
Cytogenetic risk, *n* (%)				
Favorable	24 (14.0)	28 (15.5)	18 (11.5)	70 (13.7)
Intermediate	104 (60.5)	112 (61.9)	106 (67.5)	322 (63.1)
Adverse	44 (25.6)	41 (22.7)	33 (21)	118 (23.1)
*FLT3*-ITD mutation, *n* (%)				
No	87 (76.3)	107 (79.3)	101 (77.1)	295 (77.6)
Yes	27 (23.7)	28 (20.7)	30 (22.9)	85 (22.4)
*NPM1* mutation, *n* (%)				
No	74 (75.5)	81 (66.4)	88 (66.7)	243 (69)
Yes	24 (24.5)	41 (33.6)	44 (33.3)	109(31)
*CEBPA* mutation, *n* (%)^b^				
No	65 (83.3)	72 (88.9)	74 (90.2)	211 (87.6)
Yes	13 (16.7)	9 (11.1)	44 (9.8)	30 (12.4)

*IQR* interquartile range, *WBC* white blood cell count

^a^Of non-deceased patients

^b^According to ELN 2010 classification

**Table 2:** Outcome of younger (<60 years) AML patients

	2000–2004	2005–2009	2010–2014	*P*
	*N* = 173	*N* = 181	*N* = 159	
Day-60 death, *n* (%)	16 (9.2)	15 (8.3)	7 (4.4)	0.207
CR1, *n* (%)	141 (81.5)	148 (81.8)	137 (86.2)	0.449
Induction failure, *n* (%)	18 (10.4)	22 (12.2)	14 (8.8)	0.603
Allo-SCT in CR1, *n* (%)	43 (30.5)	57 (38.5)	71 (51.8)	0.001
Auto-SCT in CR1, *n* (%)	41 (29.1)	20 (13.5)	3 (2.2)	<0.001
CR2, *n* (%)	20/68^a^ (29.4)	30/60^a^ (50.0)	22/47^a^ (46.8)	0.040
Allo-SCT in CR2, *n* (%)	0(0)	1 (3.3)	9 (40.9)	<0.001
Clinical trial, *n* (%)	98 (56.6)	46 (25.4)	49 (30.8)	<0.001
Median DFS, months (IQR)	25.1 (8.3-NR)	23.5 (7.5-NR)	43.4 (11.9-NR)	0.381
Median OS, months (IQR)	24.4 (8.7-NR)	26.6 (9.1-NR)	NR (13.4-NR)	0.035

*CR1* first complete response, *CR2* second complete response, *DFS* disease-free survival, *IQR* interquartile range, *OS* overall survival, *SCT* stem cell transplantation

^a^Relapses after CR1

**Table 3:** Multivariate analysis for overall survival in younger (<60 years) AML patients

	HR	95% CI	*P*
2005–2009	0.94	0.72–1.24	0.676
2010–2014	0.66	0.49–0.91	0.011
Age ≥50 years	1.27	1.00–1.62	0.057
Secondary AML	1.91	1.43–2.57	<0.001
*Cytogenetic risk*			
Intermediate	3.09	1.83–5.21	<0.001
Adverse	5.37	3.04–9.47	<0.001
WBC > 50 giga/L	1.58	1.18–2.10	0.002
Allo-SCT in CR1	0.66	0.49–0.89	0.006

*CI* confidence interval, *CR1* first complete response, *HR* hazard ratio, *SCT* stem cell transplantation, *WBC* white blood cell count

### Outcome of older (≥60 years) patients

**Fig. 2:**
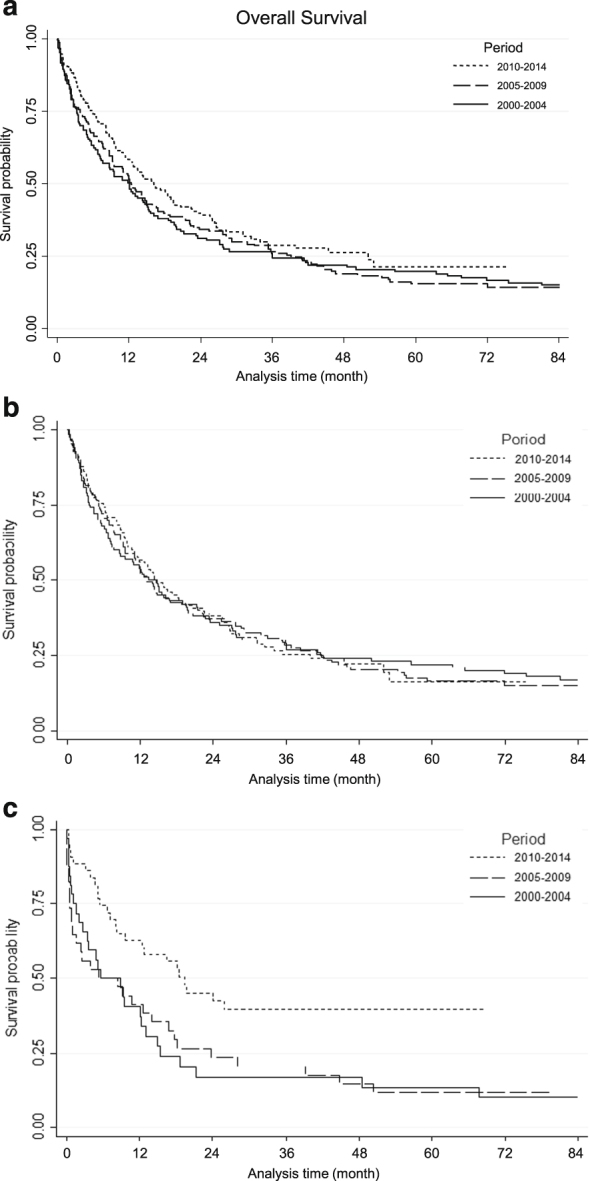
**a** Overall survival in the whole cohort of AML patients ≥60 years **b** in patients ≥60 years with WBC ≤ 50 giga/L **c** and in patients ≥60 years with WBC > 50 giga/L

The characteristics of the 463 older patients were well balanced during the study period (Supplementary Table 4). Median follow-up of patients still alive was 52.5 months (84.0, 70.6, and 35.6 months for 2000–2004, 2005–2009, and 2010–2014, respectively). Table 4 shows response to induction, treatment distribution and main outcomes. During the induction phase, the 60-day death rate was reduced from 15.8% in 2000–2004 to 10.2% in 2010–2014 (*P* = 0.196). This trend was also observed in multivariate analysis without reaching statistical significance (OR 0.61, 95% CI 0.30–1.24; *P* = 0.173) (Supplementary Table 5). There was a trend for a higher CR rate in the 2010–2014 period (74.9% vs. 66.9 and 65.6% in 2000–2004 and 2005–2009, respectively). In multivariate analysis, there was a significant interaction between the period and the WBC count indicating a significant period effect only in patients with WBC > 50 giga/L. Indeed, the probability to reach CR was significantly higher in these patients in the 2010–2014 period compared with 2000–2004 (OR 3.90, 95% CI 1.30–11.7; *P* = 0.015) (Table 5). Similar to younger patients, the number of patients receiving allogeneic stem cell transplantation significantly increased over time in both first and second CRs (Table 4). In patients reaching first CR, the cumulative incidence of death without relapse was 14% (95% CI, 10–18) and was not significantly associated with the period (*P* = 0.700). The CIR was 68% (95% CI, 62–73) and not associated with the period (*P* = 0.980) even after adjustment (Supplementary Table 6). There was no difference in DFS (Table 4 and Supplementary Table 6) and OS over time (Fig. 2a). Finally, there was a significant interaction between the period and the WBC count in the multivariate analysis for OS meaning that the 2010–2014 period had an impact only in patients with WBC > 50 giga/L independently of allogeneic stem cell transplantation (HR 0.41, 95% CI 0.24–0.71; *P* = 0.002) (Table 6 and Fig. 2b-c). There was no significant interaction between the study period and the other independent variables in the final model indicating that the period effect was not significantly different according to AML status, age, or cytogenetic risk. However, to illustrate the impact of the study period according to cytogenetics risk, age (< vs. ≥70 years) and AML status (de novo vs. secondary), Kaplan–Meier survival curves are shown in Supplementary Fig. 3 A–C.

**Table 4:** Outcome of older (≥60 years) AML patients

	2000–2004	2005–2009	2010–2014	*P*
	*N* = 133	*N* = 163	*N* = 167	
Day-60 death, *n* (%)	21 (15.8)	27 (16.6)	17 (10.2)	0.196
CR1, *n* (%)	89 (66.9)	107 (65.6)	125 (74.9)	0.150
Induction failure, *n* (%)	23 (17.3)	30 (18.4)	23 (13.8)	0.498
Allo-SCT in CR1, *n* (%)	0	7 (6.5)	24 (19.2)	<0.001
Auto-SCT in CR1, *n* (%)	0	2 (1.9)	0	0.187
CR2, *n* (%)	10/60^a^ (16.7)	12/70^a^ (17.1)	13/65^a^(20)	0.868
Allo-SCT in CR2	0	0	5 (38.5)	0.007
Clinical trial, *n* (%)	51 (38.3)	38 (23.3)	54 (32.3)	0.018
Median DFS, months (IQR)	14.3 (5.1–52)	14.2 (6.7–44)	12.7 (6.3-NR)	0.945
Median OS, months (IQR)	12.1 (3.3–36)	12.3 (3.8–39)	16.1 (5.9–52)	0.256

*CR1* first complete response, *CR2* second complete response, *DFS* disease-free survival, *IQR* interquartile range, *OS* overall survival, *SCT* stem cell transplantation

^a^Relapses after CR1

**Table 5:** Multivariate analysis for complete response in older (≥60 years) AML patients

	OR	95% CI	*P*
*Patients with WBC ≤ 50 giga/L*			
2005–2009	0.81	0.44–1.48	0.485
2010–2014	0.84	0.45–1.54	0.568
Age ≥ 70 years	0.68	0.43–1.05	0.085
Secondary AML	0.42	0.26–0.68	<0.001
Cytogenetic risk			
Intermediate	0.30	0.06–1.36	0.117
Adverse	0.14	0.03–0.66	0.013
*Patients with WBC > 50 giga/L*			
2005–2009	0.58	0.21–1.59	0.292
2010–2014	3.90	1.30–1.7	0.015
Age ≥ 70 years	0.68	0.43–1.05	0.085
Secondary AML	0.42	0.26–0.68	<0.001
*Cytogenetic risk*			
Intermediate	0.30	0.06–1.36	0.117
Adverse	0.14	0.03–0.66	0.013

*CI* confidence interval, *OR* odds ratio, *WBC* white blood cell count

**Table 6:** Multivariate analysis for overall survival in older (≥60 years) AML patients

	HR	95% CI	*P*
*Patients with WBC* ≤ *50 giga/L* ^a^			
2005–2009	1.11	0.83–1.48	0.497
2010–2014	1.17	0.87–1.59	0.304
Age ≥ 70 years	1.36	1.09–1.69	0.006
Secondary AML	1.53	1.20–1.94	0.001
Cytogenetic risk			
Intermediate	2.60	1.28–5.27	0.008
Adverse	4.74	2.28–9.89	<0.001
*Patients with WBC > 50 giga/L* ^a^			
2005–2009	1.05	0.62–1.76	0.861
2010–2014	0.41	0.24–0.71	0.002
Age ≥ 70 years	1.36	1.09–1.69	0.006
Secondary AML	1.53	1.20–1.94	0.001
Cytogenetic risk			
Intermediate	2.60	1.28–5.27	0.008
Adverse	4.74	2.28–9.89	<0.001

*CI* confidence interval, *HR* hazard ratio, *WBC* white blood cell count

^a^Allogeneic stem cell transplantation in first complete response was not independently associated with overall survival in older (≥60 years) AML patients

## Discussion

This analytic study shows that the survival of younger adult patients with AML and at least a subset of older patients, has improved in the recent period. This improvement is likely to be multifactorial. Indeed, progresses have been observed in each phase of the therapeutic course in younger AML patients, including less early deaths during induction, more allogeneic stem cell transplantation without increased NRM and more second remissions. It is plausible that the combination of all these factors resulted in survival improvement although it remains challenging to weight the impact of each phase of the treatment course. It can be easily speculated that the significant increase of allogeneic stem cell transplantations could have the higher impact in survival improvement^26, 27^. However, the period effect was independent of allogeneic stem cell transplantation. In older patients, significant advances remain to be made although the outcome of hyperleukocytic patients has been significantly improved.

With regards to chemotherapy regimen used over time, although the anthracycline dose intensity has been increased in 2010–2014 during induction by using daunorubicin at a dose of 90 mg/m^2^ (3 days) or idarubicin at a dose of 9 mg/m^2^ (5 days), the cumulative dose of both drugs was higher in the earlier period of the study. Indeed, we stopped using anthracyclines during consolidation from 2010^28, 29^. This suggests that both dose-intensity and timing of administration (e.g., the highest dose early in the therapeutic course) could be critical for the activity of anthracyclines. Furthermore, we and other have previously shown that a 60 mg/m^2^ dose of daunorubicin was equivalent to 90 mg/m^2^ but inferior to idarubicin^13, 30, 31^. Whether idarubicin (45 mg/m^2^) is better than daunorubicin (270 mg/m^2^) during induction only remains to be determined and is currently tested in a phase 3 trial of the ALFA/FILO French intergroup (Backbone InterGroup-1 trial, BIG-1, ClinicalTrials.gov: NCT02416388). The cumulative dose of cytarabine slightly increased over time together with the decrease of autologous transplantations. It is noteworthy that all therapeutic regimens used in this study included a sufficient cytarabine dose according to the new ELN guidelines^32, 33^. In 2010–2014, we have reduced the doses of cytarabine from 3 to 1.5 g/m^2^ only in patients of 50–60 years of age according to a MRC study showing equivalence between the two doses in term of disease control and survival^34^. The BIG-1 trial currently randomizes these two cytarabine doses during post remission therapy to confirm the MRC study in patients 18–60 years of age.

Despite early intensification of anthracycline during induction, the early death rate decreased over time in both younger and older patients. This is likely due to a better management of chemotherapy-induced aplasia rather than a better disease control since the induction failure rate did not change. New antifungal therapies are of high interest in this context and posaconazole, which has been approved for primary fungal infection prophylaxis during induction, remains one of the rare drugs to be associated with an improvement of survival in AML patients treated by intensive chemotherapy^23^. Moreover, there has been a recent reduction of mortality due to invasive aspergillosis during induction in AML^35^.

A major progress observed in the recent period, concerned patients with hyperleucocytosis. This was especially the case for older patients in whom a significant period effect was found for both response and OS. Since 2010, we use dexamethasone in patients with high WBC count. The rationale was to target leukostasis and inflammatory dysregulations often observed in those patients^36^. We have shown that the addition of dexamethasone to intensive chemotherapy was associated with a significant improvement of both disease-free and OS in hyperleukocytic AML patients^24^. Moreover, very recent preclinical studies have shown that the development of cytarabine resistance is associated with increased sensitivity to glucocorticoids indicating that our clinical results may suggest a synergistic effect of dexamethasone with chemotherapy, at least in hyperleukocytic patients^24, 37, 38^.

Autologous stem cell transplantation in first CR is still advocated by some investigators and included as a therapeutic option in the ELN guidelines^39^. It is suggested that autologous transplantation could benefit to favorable or intermediate-risk AML patients especially those with negative minimal residual disease. We have progressively abandoned this strategy in first CR along with molecular stratification and extension of allogeneic stem cell transplantation indications (i.e., favorable risk treated by cytarabine; others allocated to allogeneic transplantation whenever possible). Although, our strategy was associated with a better survival in the 2010–2014 period, we acknowledge that the exact role of autologous transplantation remains to be prospectively reassessed in selected patients with time-dependent statistical analyses^40^.

The main limitation of our study is its retrospective design. Our database is prospectively annotated since 2007 with follow-up for all patients since our center is the sole accredited for induction chemotherapy and allogeneic stem cell transplantation in our Region^41^. It is also possible that some of our routine therapeutic management may not be applied in other centers.

Over time, we have optimized the chemotherapy’s backbone, the management of adverse events or specific situations, such as hyperleucocytosis and extended the indications to allogeneic stem cell transplantation. It will be challenging to further improve OS of AML patients with current therapeutic options. We hope that the next major improvements will be achieved in a near future with the arrival of new potent drugs with different mechanism of action^42^.

## Electronic supplementary material

Supplementary Figure 1

Supplementary Figure 2

Supplementary Figure 3

Supplementary Table 1

Supplementary Table 2 to 6
