# Supplementary material for: Improved outcome for AML patients over the years 2000–2014
Source: Blood Cancer J. 2017 Nov 29;7(12):635. doi: 10.1038/s41408-017-0011-1 (PMC5802565; doi:10.1038/s41408-017-0011-1)

Supplementary Figure 1

A. OS of the 976 AML patients according to cytogenetic risk

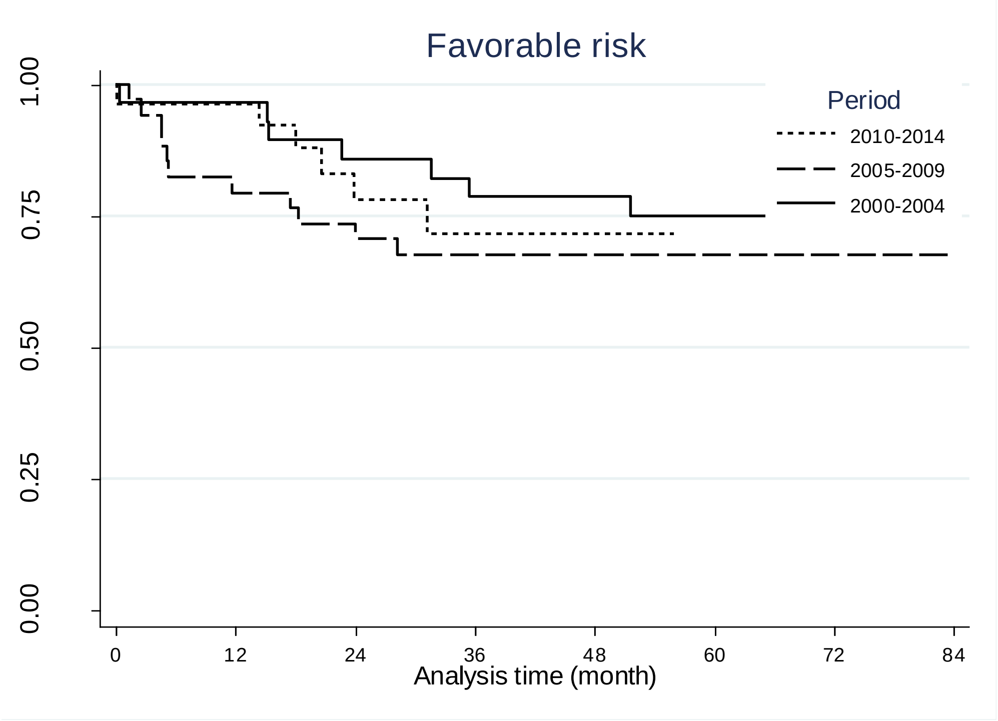

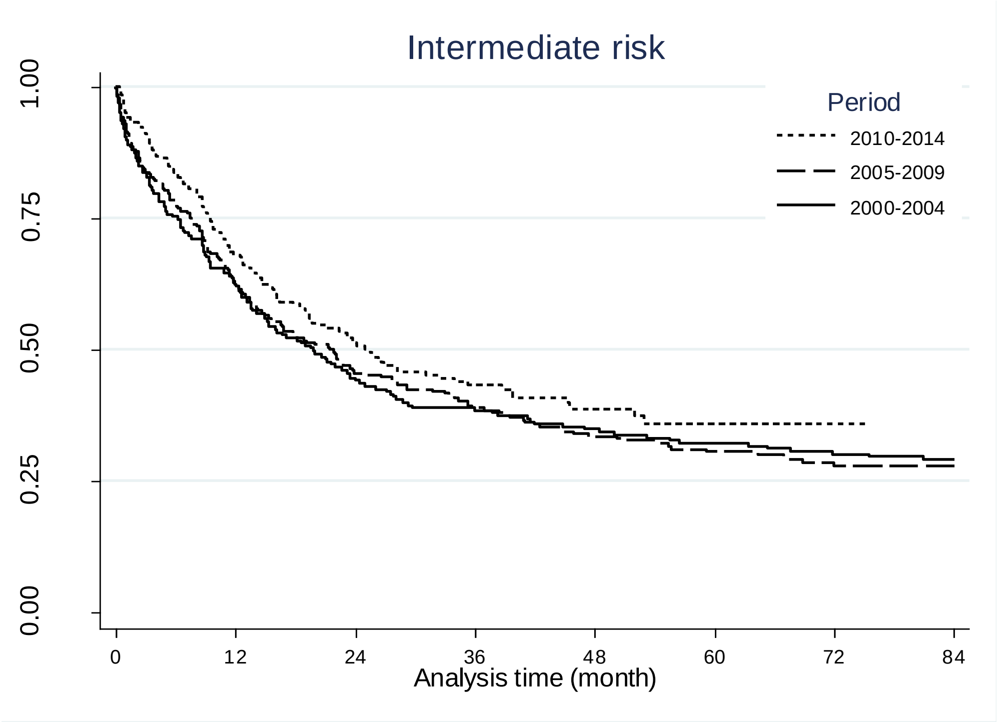

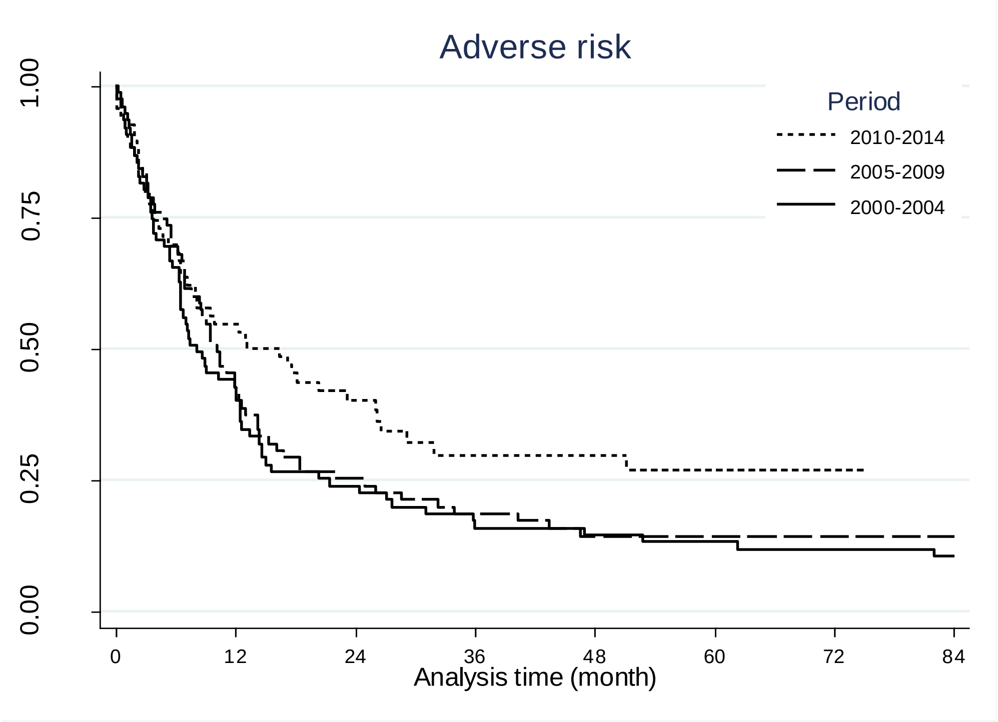
Adverse risk
Period
2010-2014
2005-2009
2000-2004
1.00
0.75
0.50
0.25
0.00
0
12
24
36
48
60
72
84
Analysis time (month)

B. OS according to AML status (de novo, secondary and therapy related)

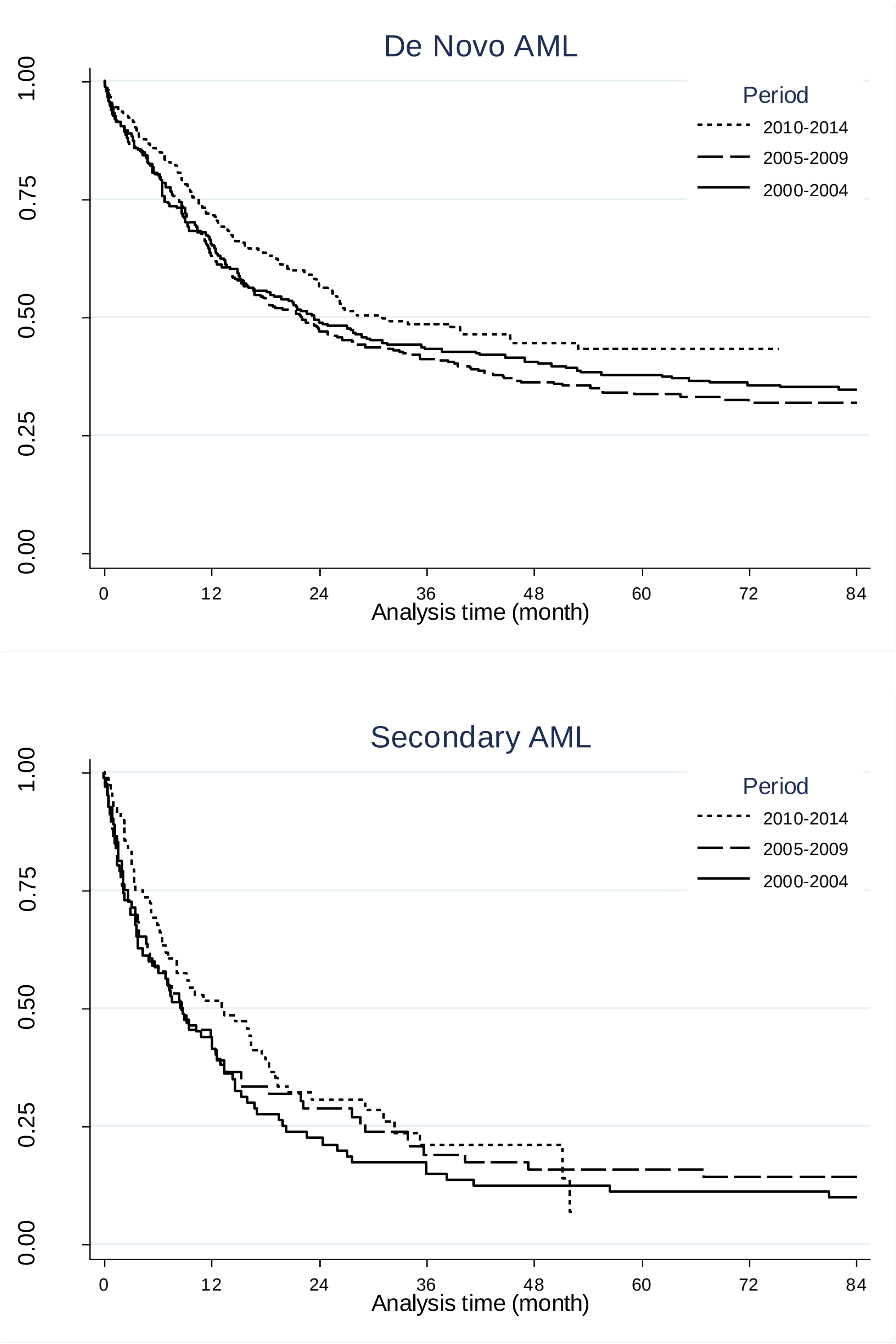

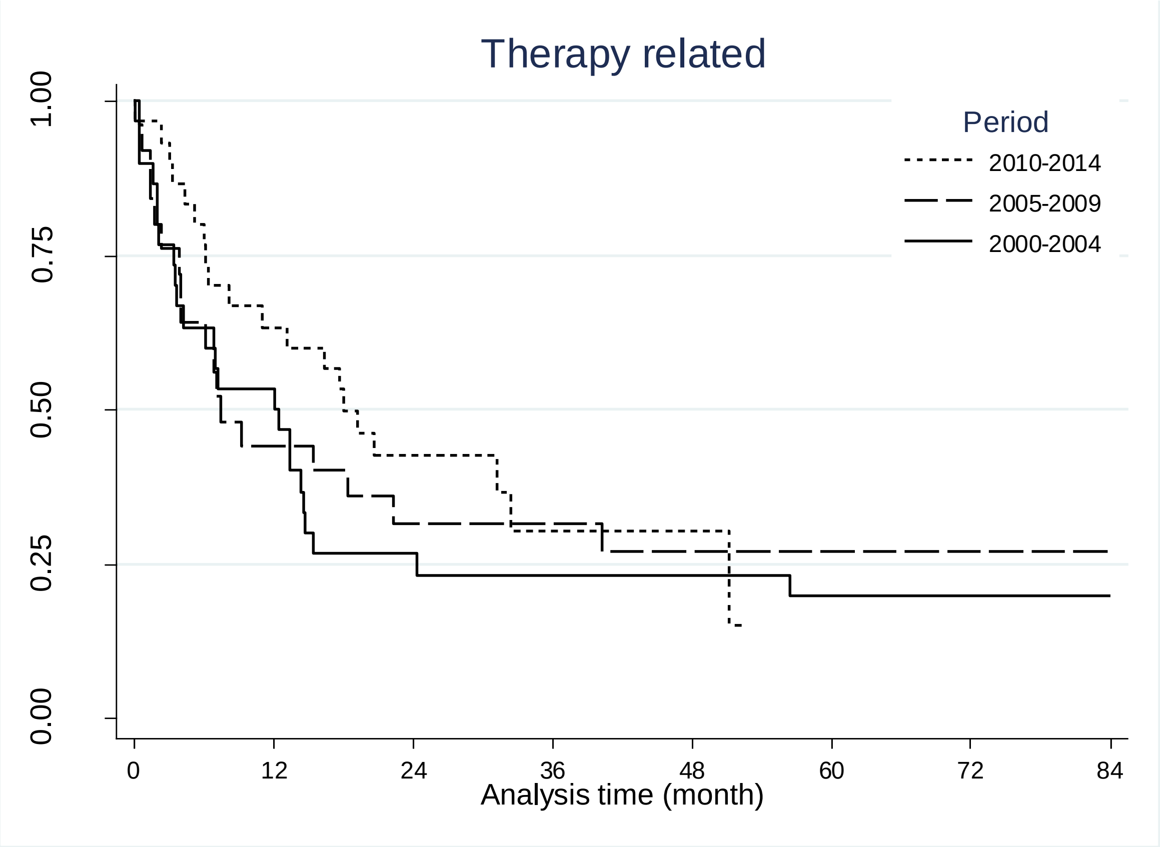

Therapy related
Period
2010-2014
2005-2009
2000-2004
0.00
0.25
0.50
0.75
1.00
0
12
24
36
48
60
72
84
Analysis time (month)

C. OS according to NPM1 mutation (intermediate cytogenetic-risk subgroup)

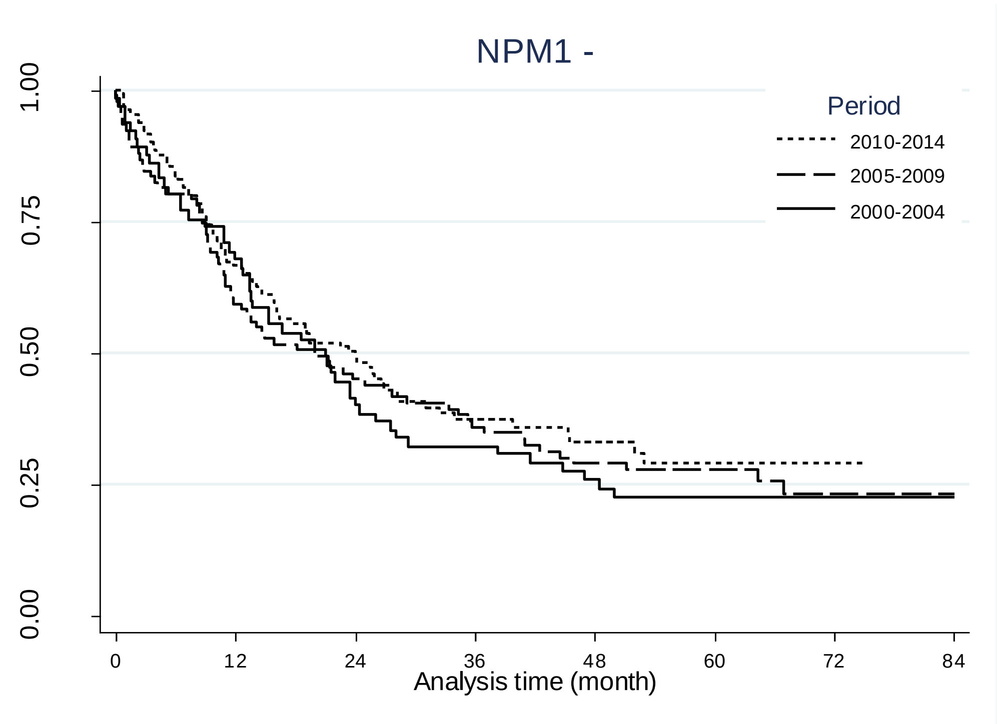

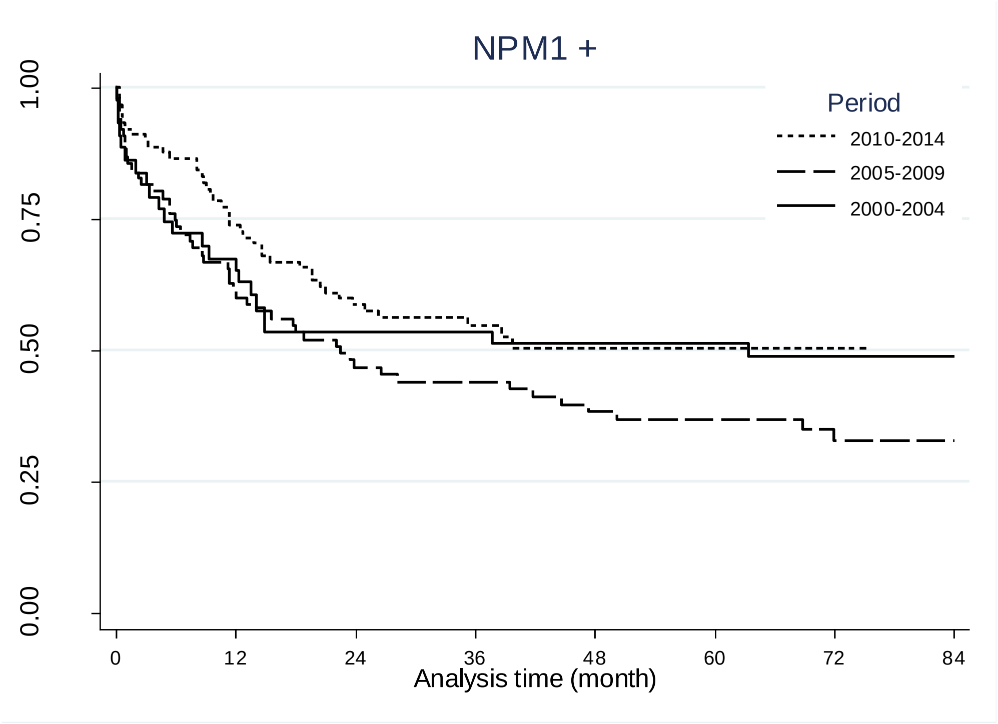

D. OS according to FLT3-ITD mutation (intermediate cytogenetic-risk subgroup)

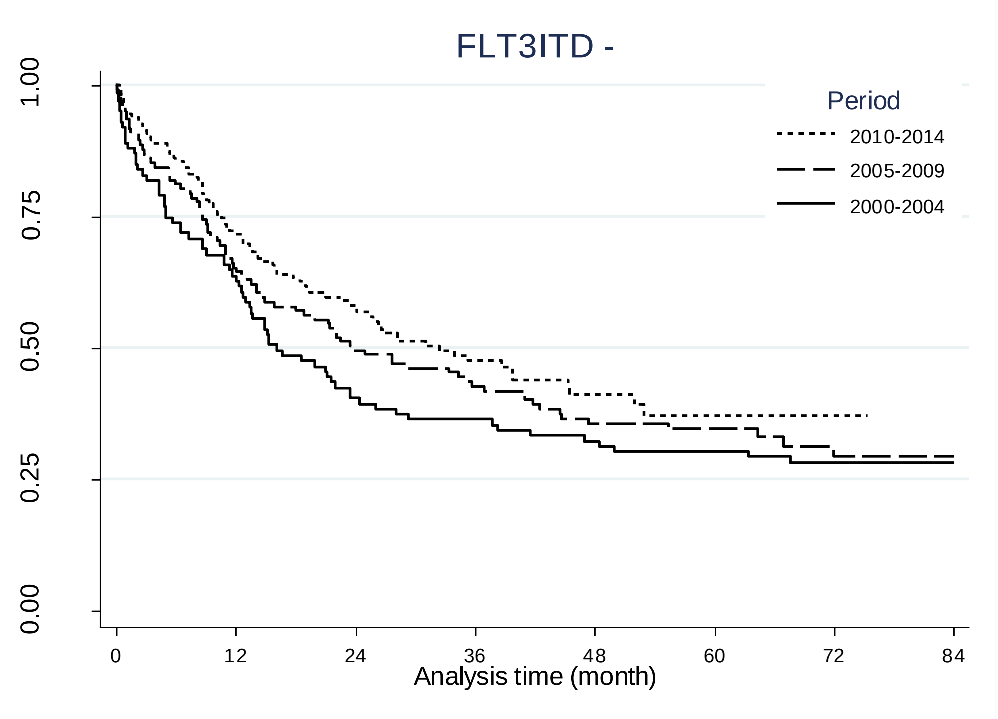

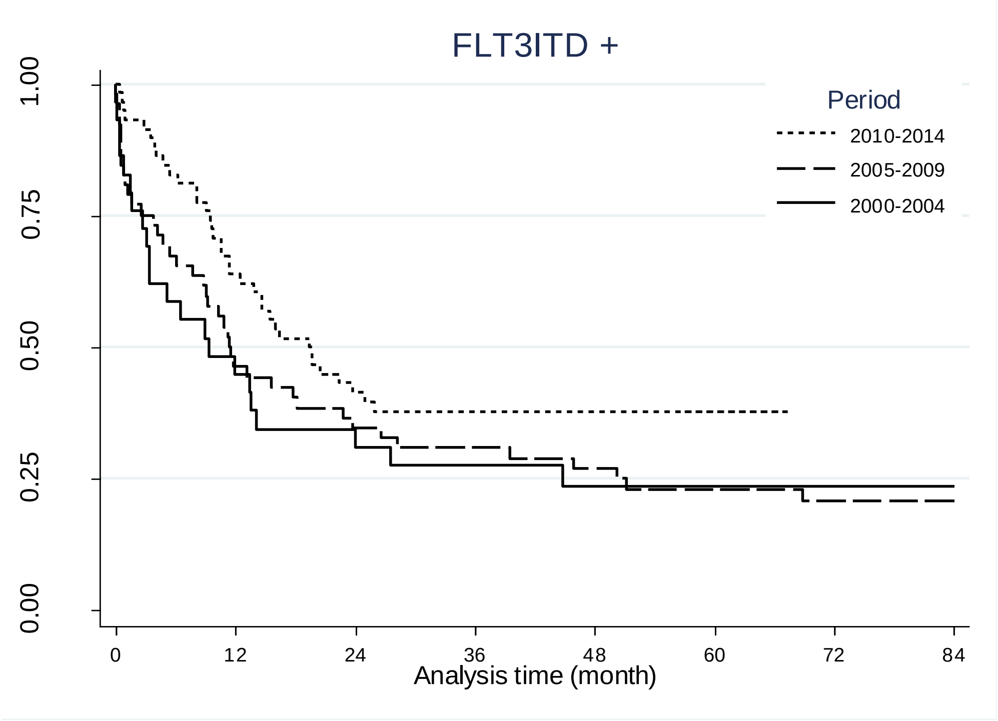

E. OS according to CEBPA mutation (intermediate cytogenetic-risk subgroup)

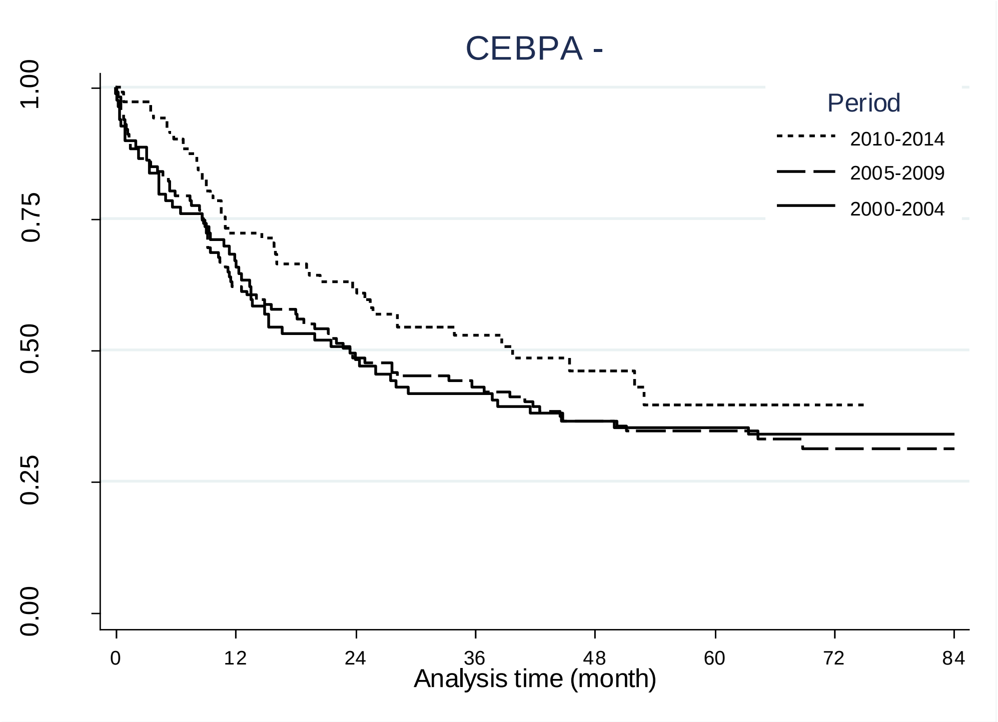

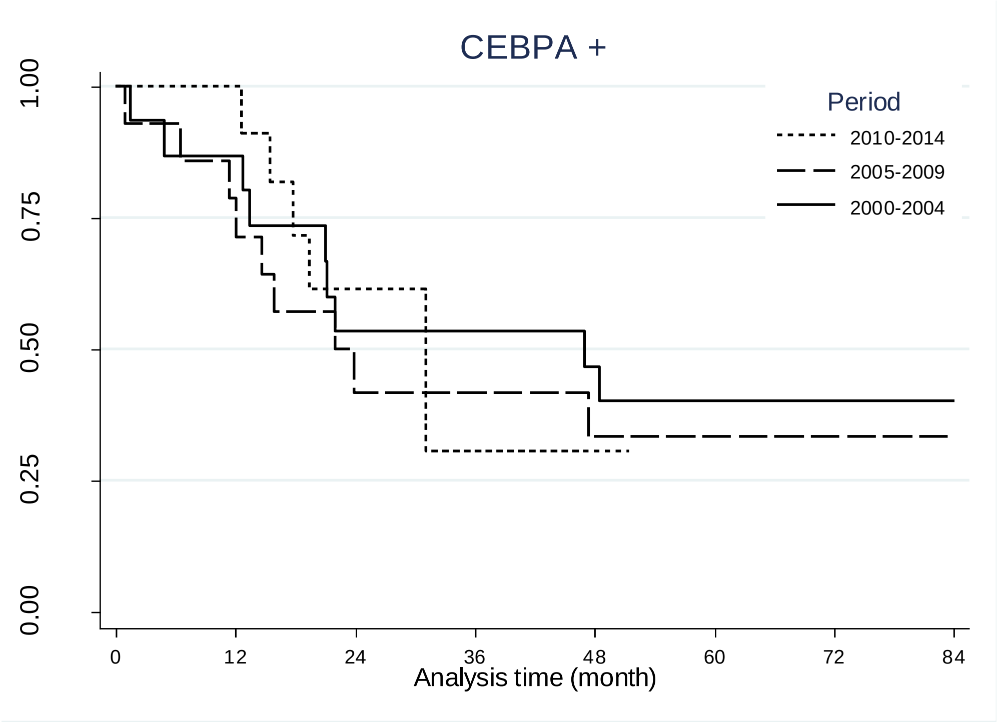

F. OS according to NPM1 mutation (normal karyotype subgroup)

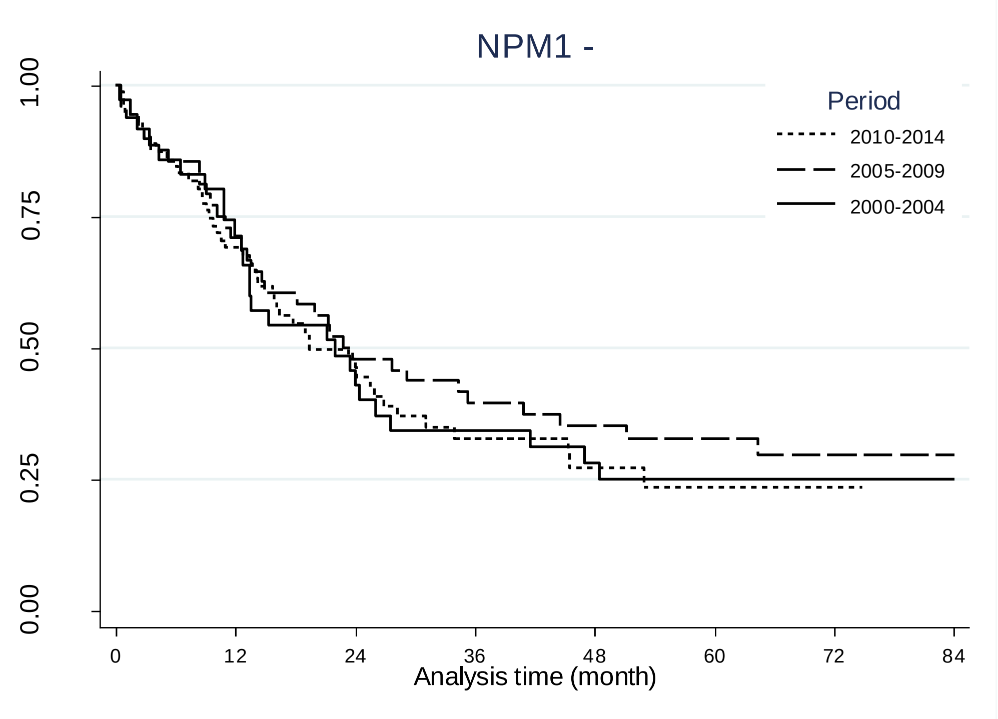

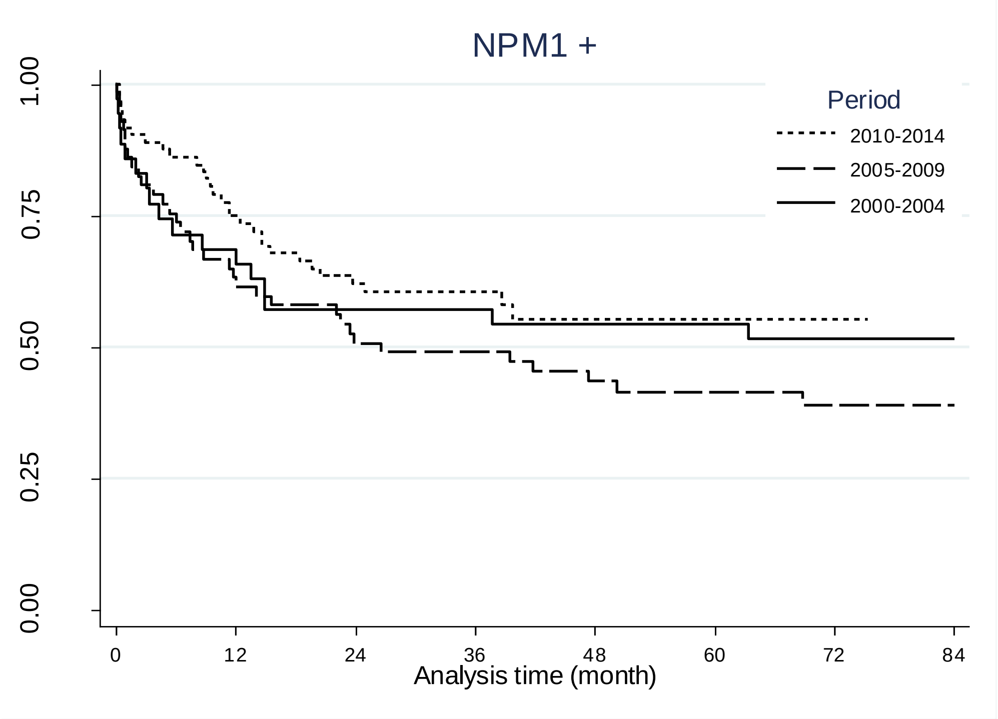

G. OS according to FLT3-ITD mutation (normal karyotype subgroup)

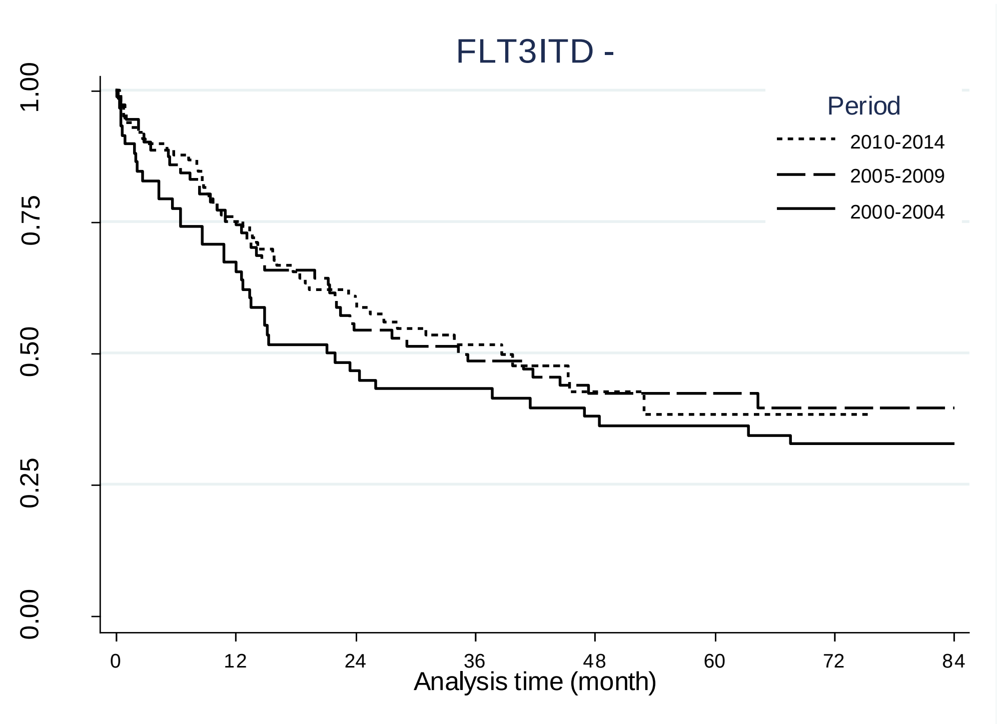

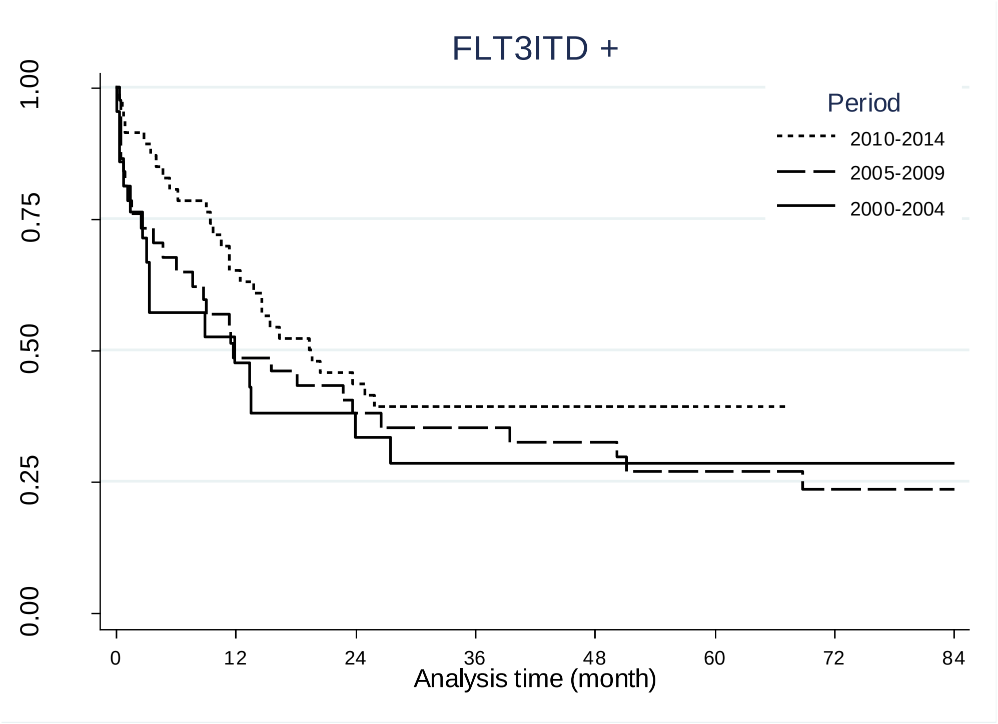

H. OS according to CEBPA mutation (normal karyotype subgroup)

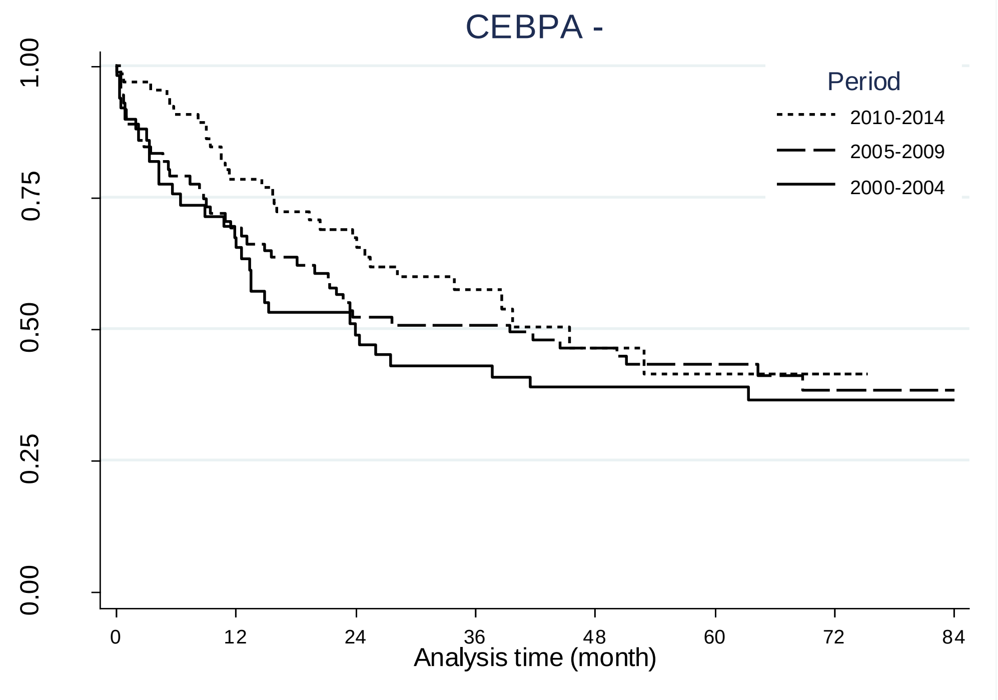

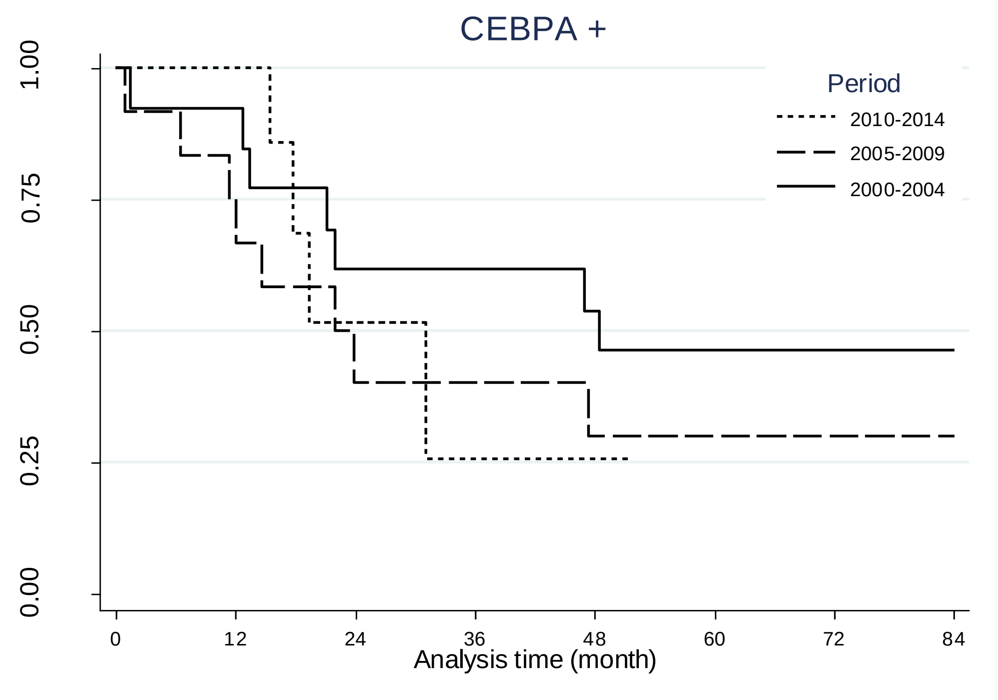

Supplement: Supplementary file 1 — Supplementary Figure 1 [file 41408_2017_11_MOESM1_ESM.pdf]
