# Supplementary material for: Improved outcome for AML patients over the years 2000–2014
Source: Blood Cancer J. 2017 Nov 29;7(12):635. doi: 10.1038/s41408-017-0011-1 (PMC5802565; doi:10.1038/s41408-017-0011-1)

**Supplementary Figure 2A:** OS according to cytogenetic risk in younger patients

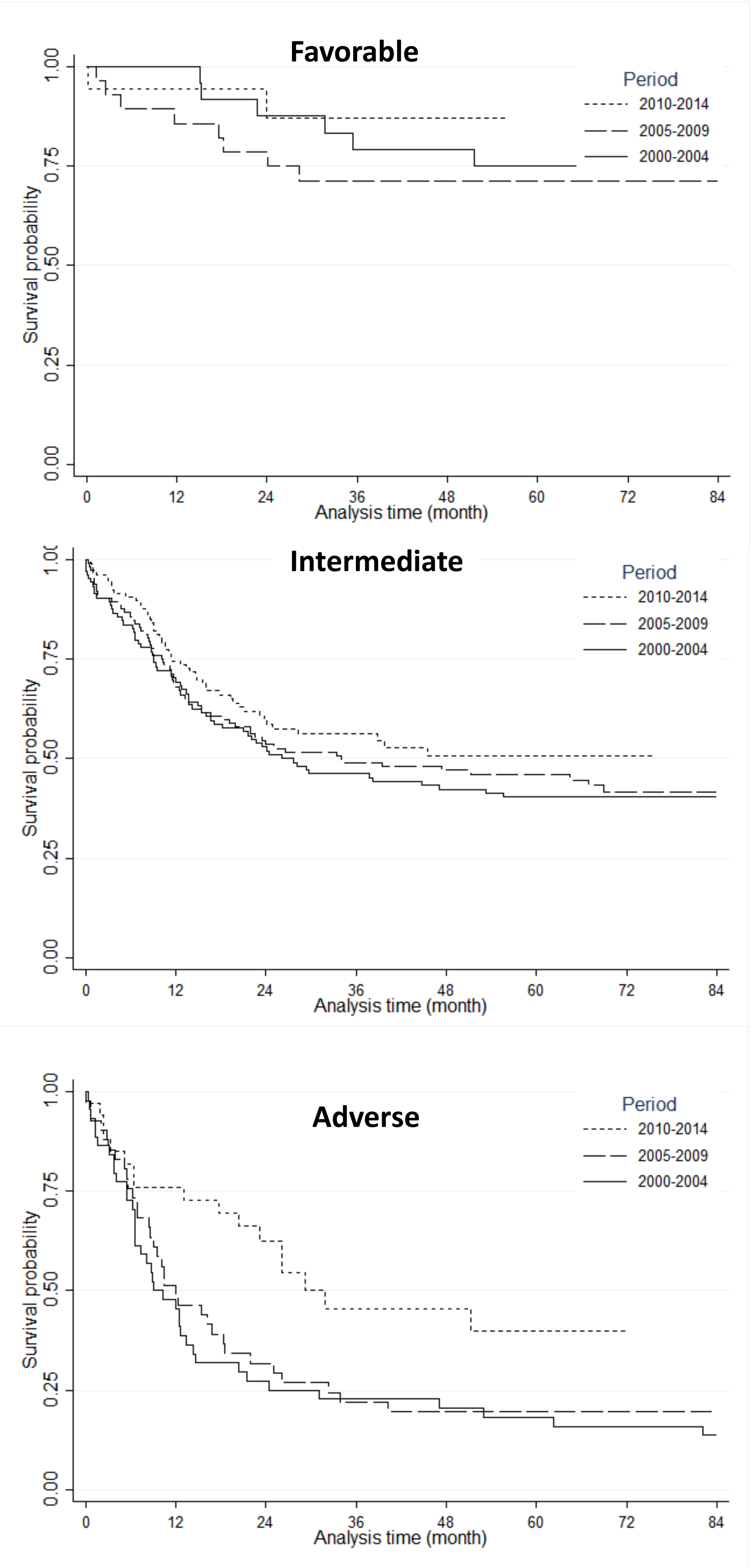

**Supplementary Figure 2B:** OS according to WBC count in younger patients

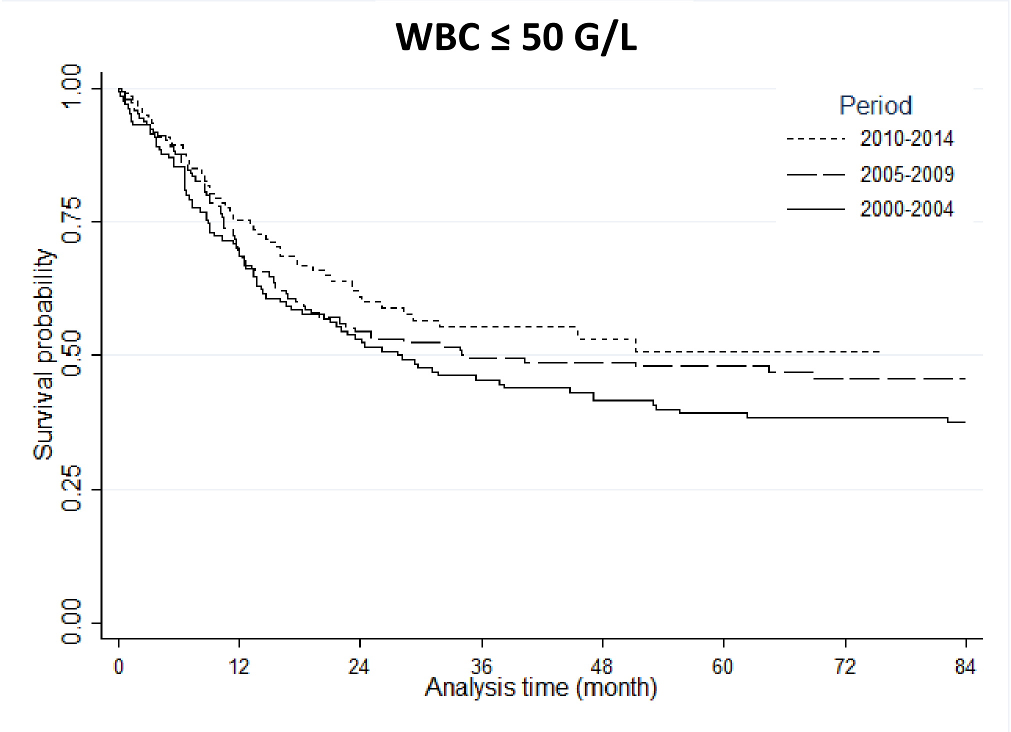


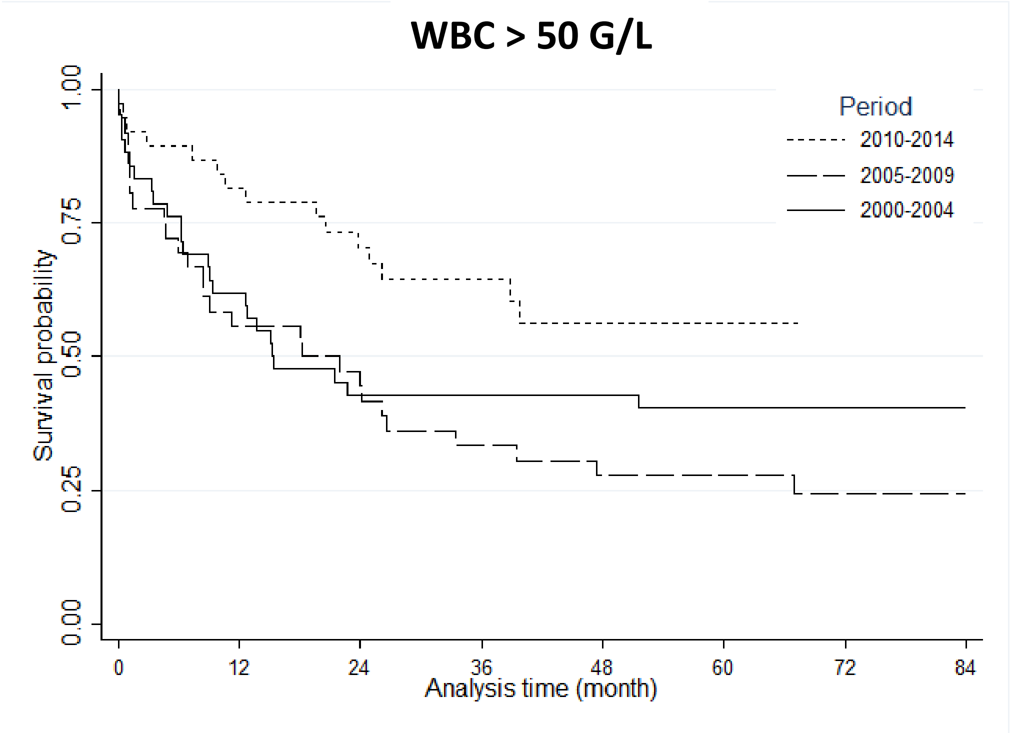

**Supplementary Figure 2C:** OS according to age (< versus ≥50y) in younger patients

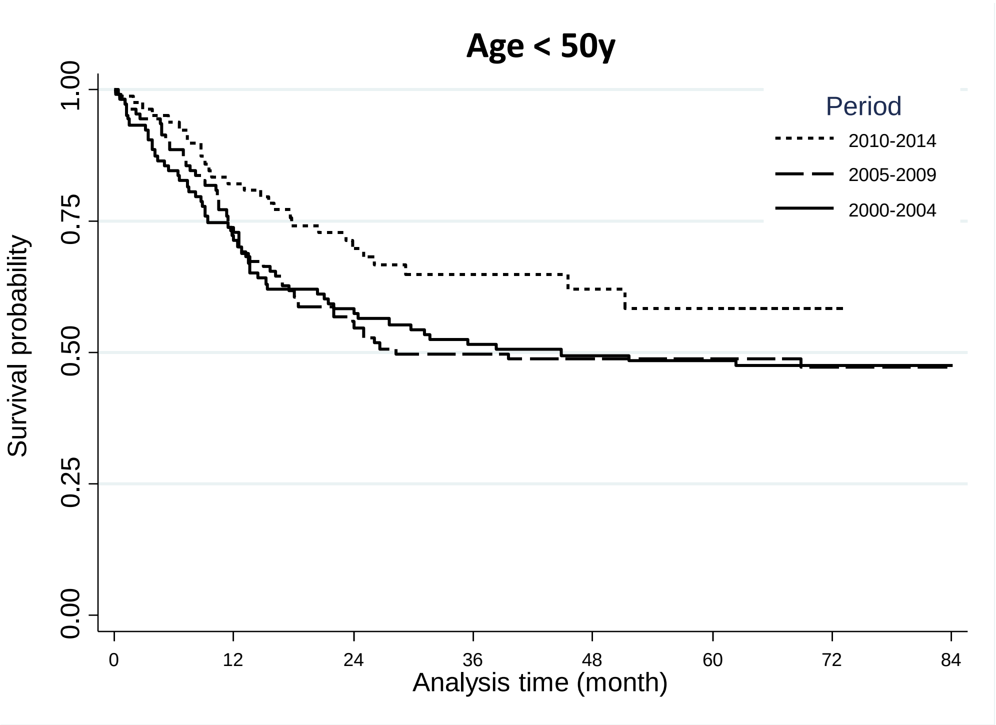


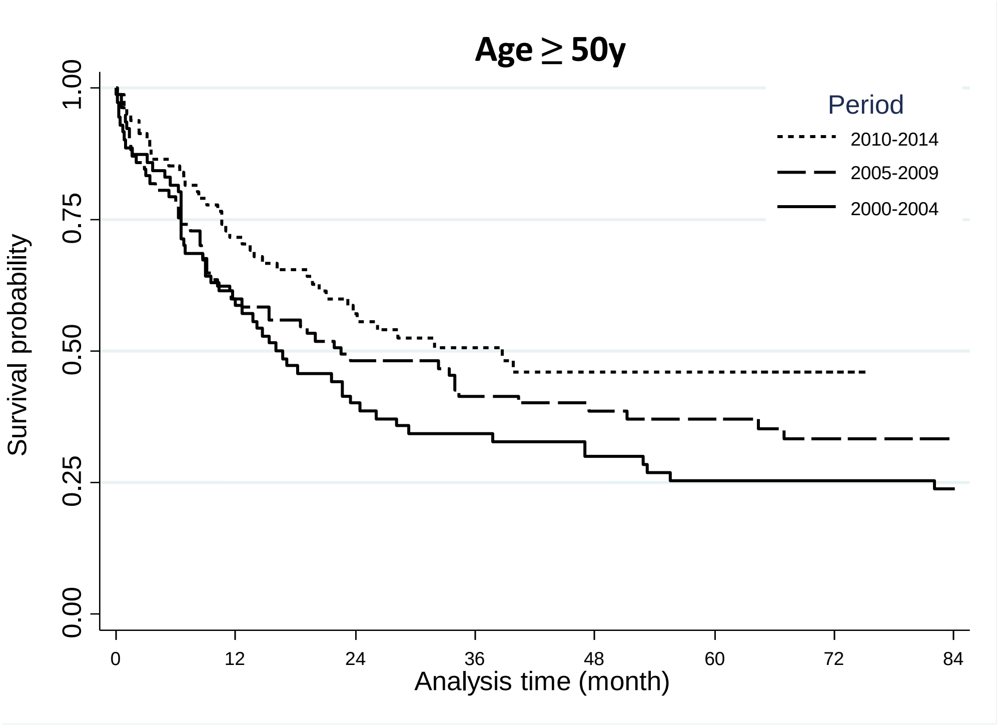

**Supplementary Figure 2D:** OS according to AML status in younger patients

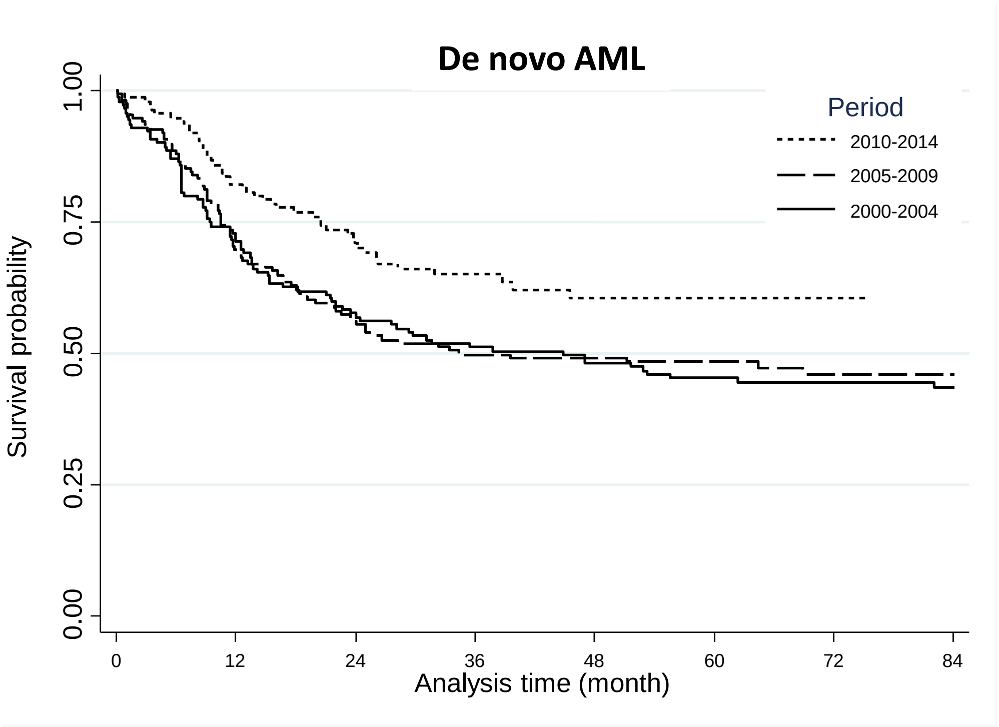


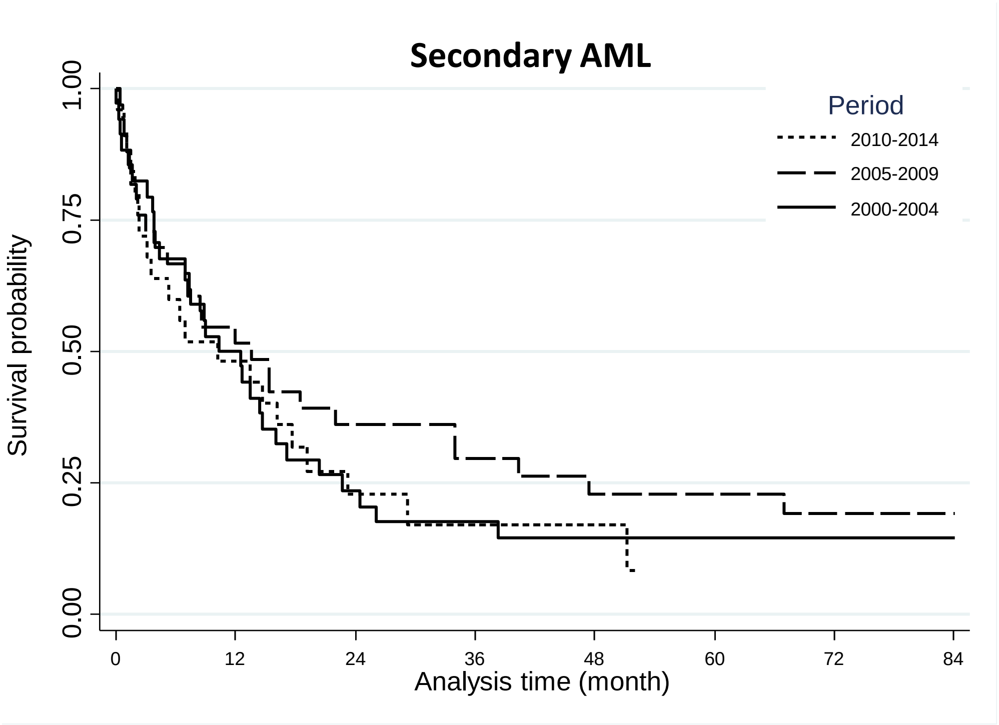

Supplement: Supplementary file 2 — Supplementary Figure 2 [file 41408_2017_11_MOESM2_ESM.pdf]
