# Supplementary material for: Improved outcome for AML patients over the years 2000–2014
Source: Blood Cancer J. 2017 Nov 29;7(12):635. doi: 10.1038/s41408-017-0011-1 (PMC5802565; doi:10.1038/s41408-017-0011-1)

Supplementary Figure 3A: OS according to cytogenetic risk in older patients

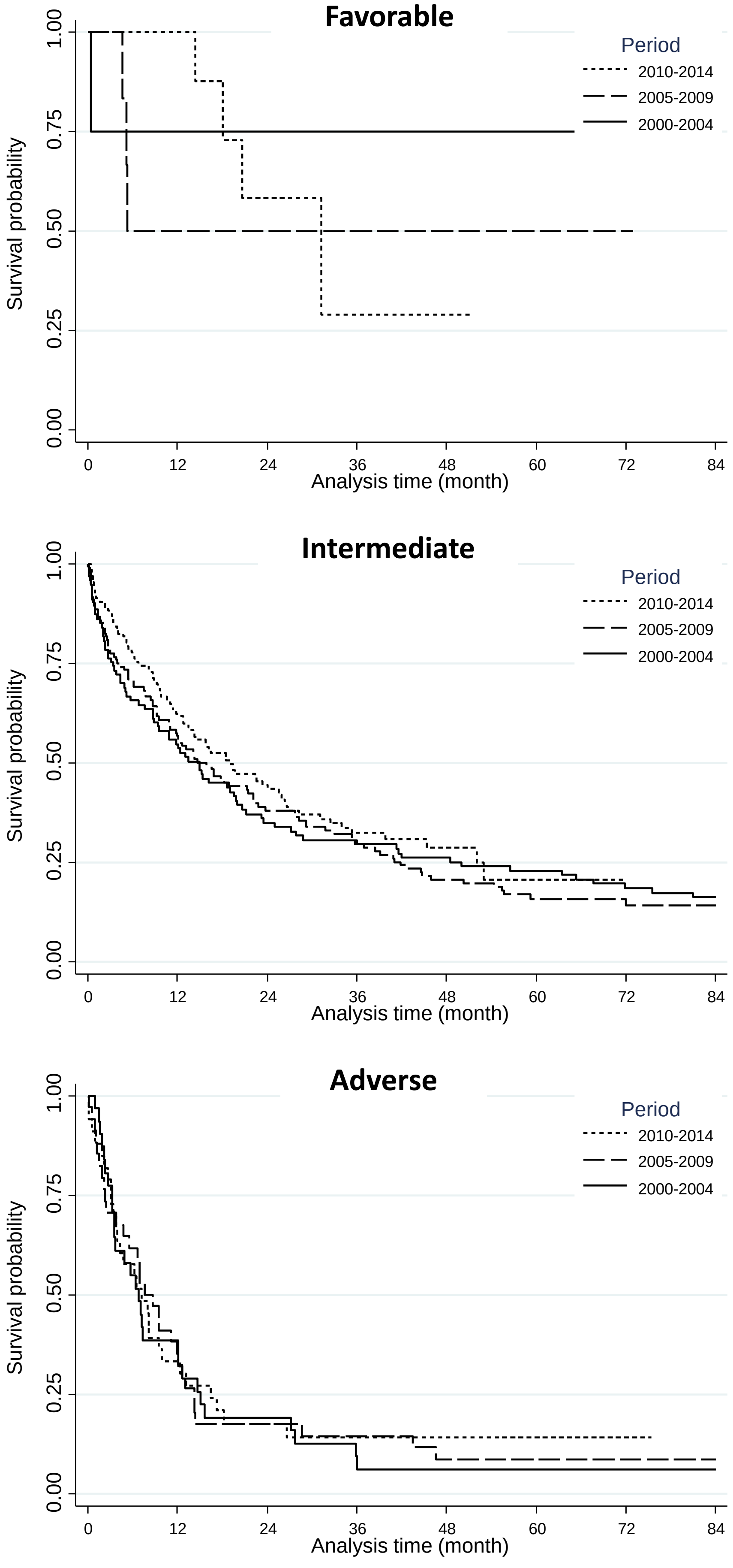

Supplementary Figure 3B: OS according to age (< versus ≥70y) in older patients

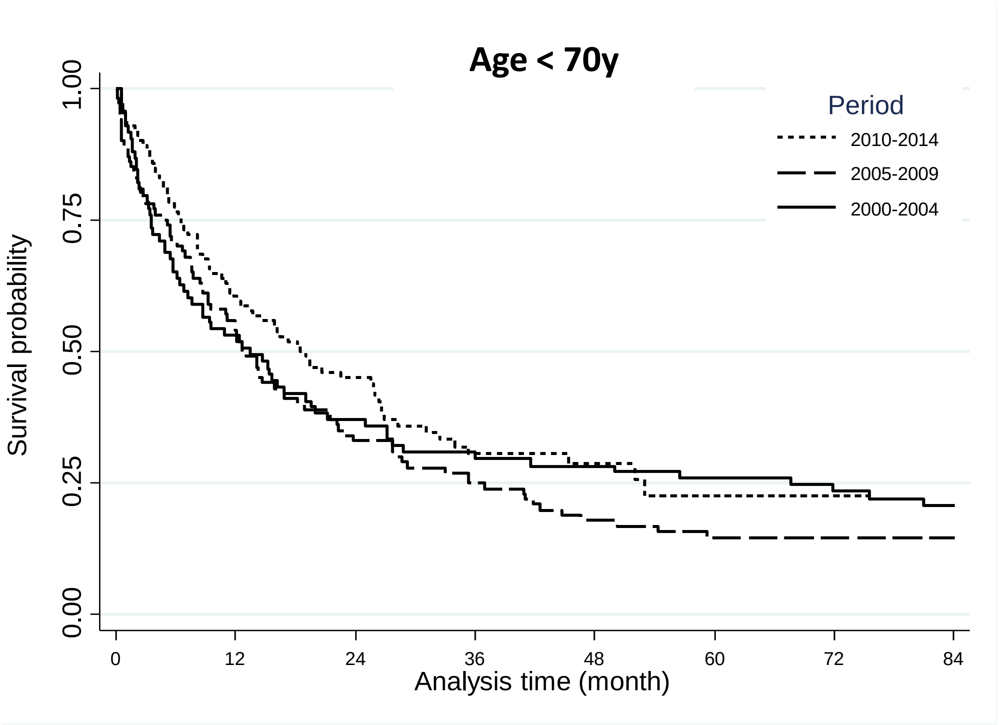

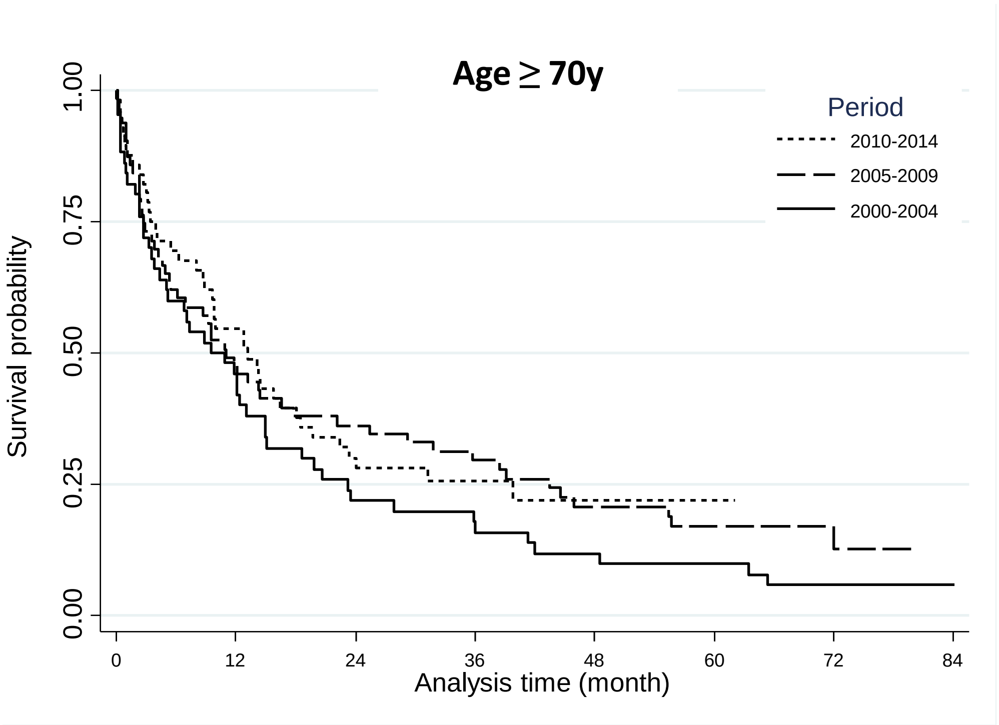

Supplementary Figure 3C: OS according to AML status in older patients

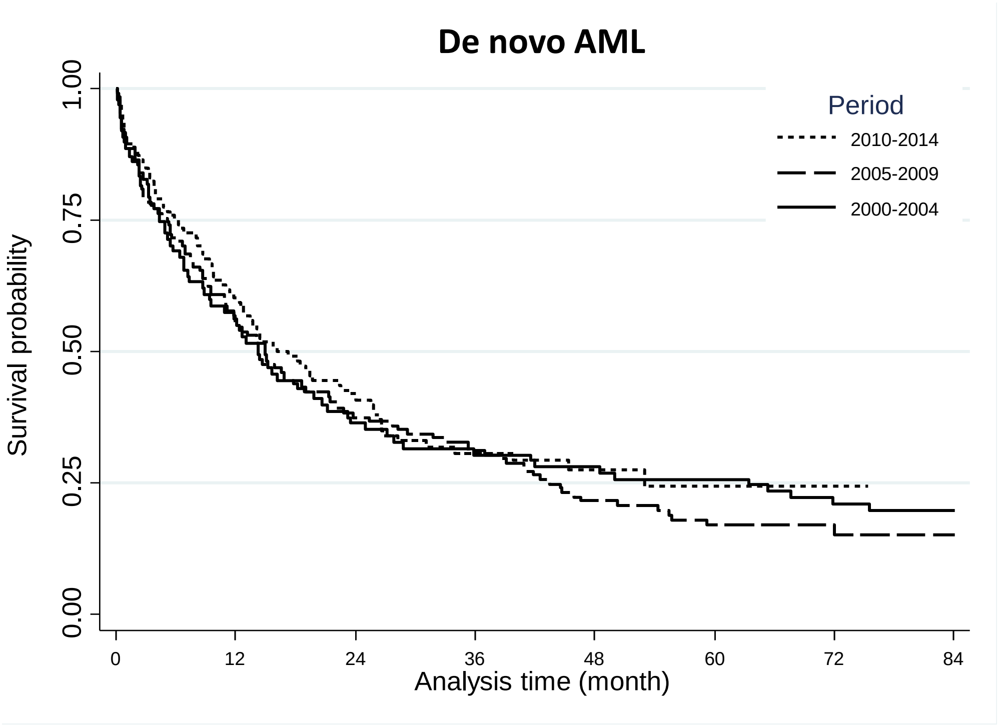

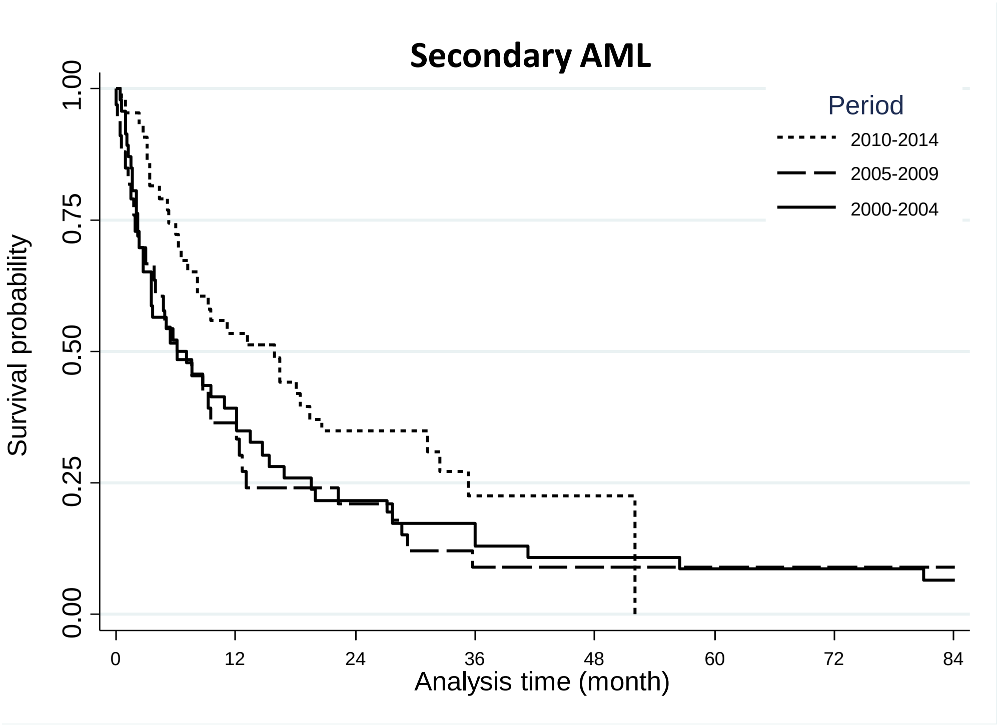

Supplement: Supplementary file 3 — Supplementary Figure 3 [file 41408_2017_11_MOESM3_ESM.pdf]
