# Supplementary material for: Improved outcome for AML patients over the years 2000–2014
Source: Blood Cancer J. 2017 Nov 29;7(12):635. doi: 10.1038/s41408-017-0011-1 (PMC5802565; doi:10.1038/s41408-017-0011-1)
Supplement: Supplementary file 4 — Supplementary Table 1 [file 41408_2017_11_MOESM4_ESM.docx]

**Supplementary Table 1: doses of chemotherapy in younger AML patients**

|  | **2000-2004 ^1-3^** | | | **2005-2009 ^4,5^** | | | **2010-2014** | | |
| --- | --- | --- | --- | --- | --- | --- | --- | --- | --- |
|  | **Induction** | **Consolidation** | **Total** | **Induction** | **Consolidation** | **Total** | **Induction** | **Consolidation** | **Total** |
| **DNR** | 60 mg/m² x3  w/o 35 x2 (d16) | C1 : 60 mg/m² x2  C2 : 60 mg/m² x2 | 420  w/o 70 mg/m² | 60 mg/m² x3  w/o 35 x2 (d16) ^4,5^  60 mg/m² x3 + 35 x2 ^5^ | 0 | 180  w/o 70 mg/m² | 90 mg/m² x3  60 mg/m² x3 + 35 x2 ^6^ |  | 270 mg/m² |
| **IDA** | 8 mg/m² x5  w/o 8 x2 (d16) | C1 : 12 mg/m² x2  C2 : 12 mg/m² x2 | 88  w/o 16 mg/m² |  |  |  | 9 mg/m² x5 |  | 45 mg/m² |
| **AMSA** |  |  |  |  | C1 : 150 mg/m² x1 | 150 mg/m² |  |  |  |
| **MIT** |  |  |  |  | C2 : 12 mg/m² x2^¶^  C3 : 12 mg/m² x2 | 24 mg/m² |  |  |  |
| **AraC** | 200 mg/m² x7  w/o 1g/m²/12h x4 (d16) | C1 : 100 mg/m² x7  C2 : 3 g/m²/12h x 4  C3* : 3 g/m²/12h x 4 | 26.1  w/o 32 g/m² | 200 mg/m² x7  w/o 1g/m²/12h x3 (d16)  500 mg/m² x3+1g/m²/12h x3 ^5^ | C1: 100 mg/m² x5  C2 : 1 g/m²/12h x 5  C3 : 1 g/m²/12h x 5  C1-C3: 3 g/m²/12h x3^5^ | 21.9  w/o 6 g/m²  61.5 g/m² ^5^ | 200 mg/m² x7  500 mg/m² x3+1g/m²/12h x3 ^6^ | C1^§^ : 1.5-3g/m²/12h x 3  C2 : 1.5-3g/m²/12h x 3  C3 : 1.5-3g/m²/12h x 3  C1-C3: 3 g/m²/12h x3 ^6^ | 28.4-55.4 g/m²  61.5 g/m² ^6^ |
| **Auto-SCT** |  | A1 : Bu-Mel140  A2: MEL 200 then BU-MEL 140 |  |  | Bu-Mel140 |  |  |  |  |
| **Allo-SCT** |  | HLA sibling  MAC (18-50y)  RIC (51-60y) |  |  | HLA sibling or  Pheno-Id 10/10  MAC (18-50y)  RIC (51-60y) |  |  | HLA sibling or  Pheno-Id 10/10  MAC (18-50y)  RIC (51-60y) |  |

* this course was given only in good-risk patients or in patients without stem-cells

^§^ 1.5 g/m² for patients 50-60y, 3g/m² for patients <50y

^4^ +/- GO 6 mg/m² at d4 LAM-IR2006 trial

^5^ CBF-AML trial

^6^ CLARA trial

1. Lioure B, Bene MC, Pigneux A, et al. Early matched sibling hematopoietic cell transplantation for adult AML in first remission using an age-adapted strategy: long-term results of a prospective GOELAMS study. *Blood*. 2012;119(12):2943-2948.

2. Chevallier P, Fornecker L, Lioure B, et al. Tandem versus single autologous peripheral blood stem cell transplantation as post-remission therapy in adult acute myeloid leukemia patients under 60 in first complete remission: results of the multicenter prospective phase III GOELAMS LAM-2001 trial. *Leukemia*. 2010;24(7):1380-1385.

3. Recher C, Bene MC, Lioure B, et al. Long-term results of a randomized phase 3 trial comparing idarubicin and daunorubicin in younger patients with acute myeloid leukaemia. *Leukemia*. 2014;28(2):440-443.

4. Hills RK, Castaigne S, Appelbaum FR, et al. Addition of gemtuzumab ozogamicin to induction chemotherapy in adult patients with acute myeloid leukaemia: a meta-analysis of individual patient data from randomised controlled trials. *Lancet Oncol*. 2014;15(9):986-996.

5. Jourdan E, Boissel N, Chevret S, et al. Prospective evaluation of gene mutations and minimal residual disease in patients with core binding factor acute myeloid leukemia. *Blood*. 2013;121(12):2213-2223.

6. Thomas X, de Botton S, Chevret S, et al. Randomized Phase II Study of Clofarabine-Based Consolidation for Younger Adults With Acute Myeloid Leukemia in First Remission. *J Clin Oncol*. 2017;35(11):1223-1230.
