# Supplementary material for: Improved outcome for AML patients over the years 2000–2014
Source: Blood Cancer J. 2017 Nov 29;7(12):635. doi: 10.1038/s41408-017-0011-1 (PMC5802565; doi:10.1038/s41408-017-0011-1)
Supplement: Supplementary file 5 — Supplementary Table 2 to 6 [file 41408_2017_11_MOESM5_ESM.docx]

**Supplementary Table 2:** Multivariate analyses for induction death, first and second complete response and induction failure in younger (<60 years) AML patients.

|  | OR | 95% CI | *P* |
| --- | --- | --- | --- |
| *Day-60 induction death* | | | |
| 2005-2009  2010-2014 | 0.89  0.43 | 0.40-1.98  0.17-1.13 | 0.780  0.089 |
| Age ≥ 50y | 2.12 | 1.00-4.49 | 0.049 |
| WBC > 50 G/L | 4.76 | 2.26-10.0 | <0.001 |
| Secondary AML | 5.41 | 2.52-11.6 | <0.001 |
| *First complete response* | | | |
| 2005-2009  2010-2014 | 0.94  1.42 | 0.53-1.68  0.75-2.68 | 0.847  0.278 |
| WBC > 50 G/L | 0.53 | 0.30-0.95 | 0.033 |
| Secondary AML | 0.23 | 0.13-0.39 | <0.001 |
| Cytogenetic risk  Intermediate  Adverse | 0.19  0.12 | 0.05-0.83  0.03-0.53 | 0.027  0.005 |
| *Induction failure* | | | |
| 2005-2009  2010-2014 | 1.31  0.93 | 0.66-2.59  0.44-1.97 | 0.444  0.857 |
| Secondary AML | 2.61 | 1.39-4.88 | 0.003 |
| Cytogenetic risk  Intermediate  Adverse | 5.91  12.1 | 0.79-44.5  1.56-94.2 | 0.084  0.017 |
| *Second complete response* | | | |
| 2005-2009  2010-2014 | 2.54  2.49 | 1.12-5.77  1.04-5.93 | 0.026  0.040 |
| Age ≥ 50y | 0.37 | 0.18-0.77 | 0.007 |
| Cytogenetic risk  Intermediate  Adverse | 0.20  0.07 | 0.06-0.71  0.02-0.26 | 0.013  <0.001 |

**Supplementary Table 3:** Multivariate analyses for relapse, non-relapse mortality, non-relapse mortality after allogeneic stem cell transplantation and disease-free survival in younger (<60 years) AML patients.

|  | HR | 95% CI | *P* |  |
| --- | --- | --- | --- | --- |
| *Cumulative incidence of relapse* | | | |  |
| 2005-2009  2010-2014 | 0.95  0.82 | 0.66-1.36  0.57-1.18 | 0.771  0.286 |  |
| WBC > 50 G/L | 1.39 | 0.97-1.98 | 0.073 |  |
| Cytogenetic risk  Intermediate  Adverse | 1.81  3.76 | 1.14-2.86  2.17-6.48 | 0.011  <0.001 |  |
| Allo-SCT in CR1 | 0.44 | 0.31-0.63 | <0.001 |  |
| *Non relapse mortality* | | | | |
| 2005-2009  2010-2014 | 1.38  1.09 | 0.69-2.76  0.52-2.29 | 0.360  0.822 |  |
| Secondary AML | 1.90 | 0.95-3.80 | 0.070 |  |
| Cytogenetic risk  Intermediate  Adverse | 4.00  4.03 | 0.94-16.9  0.88-18.6 | 0.060  0.073 |  |
| *Non relapse mortality in allografted patients* | | | | |
| 2005-2009  2010-2014 | 0.99  0.85 | 0.39-2.48  0.34-2.15 | 0.983  0.734 |  |
| Age ≥ 50y | 1.72 | 0.85-3.48 | 0.131 |  |
| WBC > 50 G/L | 0.42 | 0.15-1.21 | 0.110 |  |
| *Disease free survival** | | | | |
| 2005-2009  2010-2014 | 0.99  0.76 | 0.72-1.34  0.54-1.06 | 0.930  0.104 |  |
| Secondary AML | 1.59 | 1.11-2.29 | 0.012 |  |
| Cytogenetic risk  Intermediate  Adverse | 1.81  2.84 | 1.16-2.82  1.72-4.68 | 0.009  <0.001 |  |
| WBC > 50 G/L | 1.32 | 0.96-1.81 | 0.085 |  |

*Allogeneic stem cell transplantation in first complete response (CR1) was not independently associated with DFS in younger (<60 years) AML patients.

HR, hazard ratio; CI confidence interval; WBC, white blood cell count; SCT, stem cell transplantation; CR1, first complete response.

**Supplementary Table 4:** Characteristics of older (≥ 60 years) AML patients

|  | 2000-2004  133 (28.7%) | 2005-2009  163 (35.2%) | 2010-2014  167 (36.1%) | Total  463 (100%) |
| --- | --- | --- | --- | --- |
| Follow-up of non-deceased patients  Median, months (IQR) | 84.0 (84.0-84.0) | 70.6 (56.2-82.2) | 35.6 (24.9-49.1) | 52.5 (33.6-84.0) |
| Male, n-(%)  Female, n-(%) | 65 (48.9)  68 (51.1) | 102 (62.6)  61 (37.4) | 113 (67.7)  54 (32.3) | 280 (60.5)  183 (39.5) |
| Age, years (y)  Median (IQR)  <70y, n-(%)  ≥70y, n-(%) | 68 (64.5-72.5)  83 (62.4)  50 (37.6) | 68.7 (64-72)  100 (61.3)  63 (38.7) | 66.9 (63.4-71.9)  111 (66.5)  56 (33.5) | 68 (63.9-72)  294 (63.5)  169 (36.5) |
| AML status, n-(%)  De novo  Secondary | 87 (65.4)  46 (34.6) | 130 (79.8)  33 (20.2) | 124 (74.3)  43 (25.7) | 341 (73.7)  122 (26.3) |
| Performance status, n-(%)  0-1  2-4 | 68 (71.6)  27 (28.4) | 100 (80)  25 (20) | 123 (78.3)  34 (21.7) | 291 (77.2)  86 (22.8) |
| WBC, (giga/L)  Median, IQR  ≤50, n-(%)  >50, n-(%) | 10.7 (2.6-41.1)  100 (75.8)  32 (24.2) | 8.3 (2.7-37.6)  129 (79.1)  34 (20.9) | 11.2 (2.6-50.4)  124 (74.3)  43 (25.7) | 9.4 (2.6-42.3)  353 (76.4)  109 (23.6) |
| Cytogenetic risk, n-(%)  Favorable  Intermediate  Adverse | 4 (3.1)  93 (72.7)  31 (24.2) | 6 (3.8)  120 (75.0)  34 (21.3) | 8 (4.8)  126 (75.4)  33 (19.8) | 18 (4.0)  339 (74.5)  98 (21.5) |
| *FLT3*-ITD mutation, n-(%)  No  Yes | 66 (88)  9 (12) | 86 (74.1)  30 (25.9) | 108 (78.8)  29 (21.2) | 260 (79.3)  68 (20.7) |
| *NPM1* mutation, n-(%)  No  Yes | 27 (58.7)  19 (41.3) | 68 (65.4)  36 (34.6) | 92 (67.2)  45 (32.8) | 187 (65.2)  100 (34.8) |
| *CEBPA* mutation*, n-(%)  No  Yes | 27 (90)  3(10) | 55 (88.7)  7 (11.3) | 33 (89.2)  4 (10.8) | 187 (89.1)  14 (10.9) |

IQR, interquartile range; WBC, white blood cell count.

* according to ELN 2010 classification.

**Supplementary Table 5:** Multivariate analyses for induction death, induction failure and second complete response in older (≥ 60 years) AML patients.

|  | OR | 95% CI | *P* |
| --- | --- | --- | --- |
| *Day-60 induction death* | | | |
| 2005-2009  2010-2014 | 1.17  0.61 | 0.61-2.22  0.30-1.24 | 0.639  0.173 |
| WBC > 50 G/L | 2.92 | 1.67-5.11 | <0.001 |
| *Induction failure* | | | |
| 2005-2009  2010-2014 | 1.28  0.87 | 0.68-2.42  0.45-1.68 | 0.437  0.673 |
| Secondary AML | 2.22 | 1.30-3.81 | 0.004 |
| Cytogenetic risk  Intermediate  Adverse | 2.51  7.19 | 0.32-19.5  0.91-57.0 | 0.378  0.062 |
| *Second complete response* | | | |
| 2005-2009  2010-2014 | 1.03  1.08 | 0.38-2.82  0.39-2.99 | 0.955  0.889 |
| Age ≥ 70y | 0.12 | 0.03-0.43 | 0.001 |
| Cytogenetic risk  Intermediate  Adverse | 0.10  0.01 | 0.01-0.90  0.001-0.20 | 0.040  0.002 |

OR, Odds Ratio ; WBC, white blood cell count, CI, confidence interval.

**Supplementary Table 6:** Multivariate analyses for relapse, non-relapse mortality and disease-free survival in older (≥ 60 years) AML patients.

|  | HR | 95% CI | *P* |
| --- | --- | --- | --- |
| *Cumulative incidence of relapse* | | | |
| 2005-2009  2010-2014 | 1.12  1.20 | 0.79-1.58  0.82-1.75 | 0.530  0.350 |
| Cytogenetic risk  Intermediate  Adverse | 2.17  2.66 | 0.98-4.78  1.13-6.28 | 0.055  0.026 |
| Allo-SCT in CR1 | 0.50 | 0.27-0.92 | 0.026 |
| *Non relapse mortality** | | | |
| *Disease free survival* | | | |
| 2005-2009  2010-2014 | 1.07  1.10 | 0.78-1.47  0.79-1.54 | 0.674  0.559 |
| Cytogenetic risk  Intermediate  Adverse | 1.84  3.03 | 0.91-3.74  1.42-6.45 | 0.091  0.004 |
| Allo-SCT in CR1 | 0.61 | 0.37-1.02 | 0.060 |

* No known predictive factors including WBC count, cytogenetic risk, age and secondary AML was significantly associated with non-relapse mortality.

HR, hazard ratio; CI, confidence interval; SCT, stem cell transplantation; CR1, first complete response.
